# Functional FTSH4 complexes in *Arabidopsis* mitochondria: a megacomplex with SLP1 and SLP1-free smaller complexes

**DOI:** 10.1093/pcp/pcag006

**Published:** 2026-02-19

**Authors:** Agata Maziak, Małgorzata Heidorn-Czarna, Bernadette Gehl-Väisänen, Urszula Kaźmierczak, Hanna Jańska

**Affiliations:** Department of Cellular Molecular Biology, Faculty of Biotechnology, University of Wrocław, F. Joliot-Curie 14A, Wrocław 50-383, Poland; Department of Cellular Molecular Biology, Faculty of Biotechnology, University of Wrocław, F. Joliot-Curie 14A, Wrocław 50-383, Poland; Department of Applied Physics, Aalto University, P.O. Box 11000 (Otakaari 1B), FI-00076 Aalto, Finland; Department of Cellular Molecular Biology, Faculty of Biotechnology, University of Wrocław, F. Joliot-Curie 14A, Wrocław 50-383, Poland; Department of Cellular Molecular Biology, Faculty of Biotechnology, University of Wrocław, F. Joliot-Curie 14A, Wrocław 50-383, Poland

**Keywords:** *Arabidopsis thaliana*, FTSH4 protease, mitochondria, protein complexes, stomatin, SLP1

## Abstract

The FTSH4 protease is a major component of the *Arabidopsis* mitochondrial protein quality control system. It has both a proteolytic and a chaperone-like activity and forms complexes anchored in the inner mitochondrial membrane. Here, we show that FTSH4 assembles into two distinct forms: a dominant high-molecular-weight megacomplex with stomatin-like protein 1 (SLP1) and smaller SLP1-free assemblies. In the *slp1-1* mutant, the FTSH4–SLP1 megacomplex is absent, while the abundance of SLP1-free FTSH4 assemblies is nearly doubled. Despite this, *slp1-1* maintains wild-type levels of the FTSH4 substrates TIM17-2 and NAD9, indicating that the SLP1-free assemblies retain proteolytic activity. Furthermore, *slp1-1* mitochondria accumulate fewer detergent-resistant HSP23.6 aggregates under elevated temperature than *ftsh4-1* and even wild-type mitochondria. Consequently, the mitochondrial unfolded protein response reported in *ftsh4-1* is not induced in *slp1-1*. Although *slp1-1* plants display morphological changes previously associated with *ftsh4-1*, such as shorter inflorescence stems due to premature arrest of the shoot apical meristem, these are less pronounced. Taken together, the increased abundance of SLP1-free FTSH4 assemblies is sufficient to support general mitochondrial proteostasis, providing effective protection against heat-induced aggregation of mitochondrial proteins. In contrast, the FTSH4–SLP1 megacomplex more effectively fulfils the meristem-specific functions of FTSH4.

## Introduction

Mitochondria must ensure protein homeostasis to function correctly. ATP-dependent proteases with two different activities, proteolytic and chaperone-like, are essential to achieve this state (Janska et al. 2013, Opalinska and Janska 2018). In addition, it has been reported that ATP-dependent proteases can function as chaperones independently of their proteolytic activity (Lee and Suzuki 2008, Ghifari et al. 2023). The FTSH4 protease is one of several ATP-dependent proteases in *Arabidopsis* mitochondria (Janska et al. 2010). It is a zinc metallopeptidase that spans the inner mitochondrial membrane (IMM) and exposes its catalytic domain to the intermembrane space (IMS) (Urantowka et al. 2005) and so belongs to the conserved *i*-AAA proteases, a family of ATP-dependent proteases that target the IMS. *Arabidopsis* plants lacking FTSH4 show no abnormalities in morphology when grown under long days and optimal growth temperatures, but severe morphological alterations occur during long days under a prolonged moderately elevated temperature of 30°C (Smakowska et al. 2016, Maziak et al. 2021) or at the late developmental stage under short-day conditions (Gibala et al. 2009, Kicia et al. 2010). These conditional morphological defects of *ftsh4* plants have been correlated with oxidative stress, accumulation of detergent-insoluble mitochondrial protein aggregates, mitochondrial unfolded protein response (UPR^mt^), reduced abundance of complexes I and V, as well as decreased ATP and cardiolipin content (Gibala et al. 2009, Smakowska et al. 2016, Maziak et al. 2021). Interestingly, despite the lack of abnormalities in the overall plant morphology, in *ftsh4* mutant plants grown under optimal growth temperature, giant mitochondria coexisting with normal ones have been observed (Smakowska et al. 2016). Links between a loss of FTSH4 and defective auxin and salicylic acid signaling have also been reported (Zhang et al. 2014, 2017). Several mitochondrial proteins have been identified as proteolytic substrates of FTSH4 (Opalinska et al. 2017, 2018, Maziak et al. 2021), the best studied of which is TIM17-2, an essential component of the inner membrane TIM17:23 translocase (Opalinska et al. 2018). A lack of FTSH4 protease is associated with an increased abundance of TIM17-2 and an enhanced import of model substrates of the TIM17:23 translocase. Recently, we showed that both the proteolytic activity and the chaperone-like activity of FTSH4 are crucial for protecting mitochondrial proteins, including the heat-inducible small mitochondrial heat shock protein HSP23.6, against aggregation due to prolonged moderate heat stress (Maziak et al. 2021). The importance of the chaperone-like activity of FTSH4 under conditions of moderate heat stress is additionally supported by the partially restored wild-type (WT) phenotype of *ftsh4* plants expressing a proteolytically inactive FTSH4 (Maziak et al. 2021).

Members of the band-7 protein family, a diverse group of conserved membrane proteins involved in forming large protein complexes serving as protein–lipid scaffolds, regulate FTSH proteases in different organisms (Gehl and Sweetlove 2014). Stomatins are among the members of the band-7 protein family, and two of them, SLP1 and SLP2, have been found in *Arabidopsis*’s inner mitochondrial membrane (Gehl et al. 2014). SLP1 is present in a large, ~3 MDa complex, and its loss results in decreased protein levels and activity of mitochondrial complex I and its supercomplexes, with I_2_III_2_ (previously defined as I_2_III_4_; Braun 2020) being the most affected. These findings suggest that SLP1 plays a role in organizing respiratory supercomplexes in *Arabidopsis* mitochondria.

It has been reported that the human homolog of *Arabidopsis* FTSH4 protease, YME1L, forms an inner membrane complex with stomatin-like protein 2 (SLP2) and the rhomboid protease PARL (Wai et al. 2016). This complex, known as SPY (for SLP2–PARL–YME1L), migrates in native electrophoresis at 2 MDa and is required for the proteolytic regulation of mitochondrial dynamics and quality control. Another 1.5 MDa SPY-like complex has been discovered in the unicellular protozoan *Trypanosoma brucei* (Serricchio and Bütikofer 2021). Its two components, TbSlp2 and TbYme1, are involved in heat stress resistance and negatively regulate each other’s expression levels (Serricchio and Bütikofer 2021).

Here, we demonstrate that in *Arabidopsis* mitochondria, the FTSH4 protease (a homolog of human YME1L and *Trypanosoma* TbYme1) and SLP1 (a homolog of human SLP2 and *Trypanosoma* TbSLP2) form a megacomplex of ~3–4 MDa. FTSH4 is also found in lower-molecular-weight complexes lacking SLP1, ranging in size from ~160 kDa to 1.5 MDa. The FTSH4–SLP1 megacomplex predominates in WT mitochondria, whereas only the SLP1-free, low-molecular-weight (LMW) complexes are present in the *slp1-1* mutant. Moreover, these complexes are substantially more abundant compared with WT mitochondria, most likely due to posttranscriptional regulation. These SLP1-free LMW complexes of FTSH4 are efficient in protecting the mitochondrial protein HSP23.6 from aggregation upon moderate temperature stress but cannot fully compensate for the lack of the FTSH4–SLP1 megacomplex in the specific role of FTSH4 in the functioning of shoot and root meristems.

## Results

### FTSH4 and SLP1 interact with each other

Two stomatin-like proteins, SLP1 and SLP2, have been reported earlier among the proteins to copurify with *FTSH4*^*H486Y*^ (a proteolytically inactive FTSH4 variant) in plants grown at 22°C (Opalinska et al. 2017). Here, we repeated the trapping approach using mitochondria isolated from *FTSH4*^*H486Y*^ plants cultivated at 22°C and additionally at 30°C. Mitochondria extracted from *ftsh4-1* plants grown at the appropriate temperature were used as a control for nonspecific anti-FLAG antibody interactions. SLP1 and SLP2 were identified by mass spectrometry to copurify with *FTSH4*^*H486Y*^ at both growth temperatures (Supplementary Tables S1, S2, S6, and S7). We also conducted a reciprocal coimmunoprecipitation experiment in which formaldehyde-treated mitochondria from the WT plants (*qrt1-2*) were immunoprecipitated with an anti-SLP1 antibody, and mitochondria from an *slp1-2* mutant were used as a control (Supplementary Tables S3 and S8). As expected, FTSH4 was identified using mass spectrometry among copurifying proteins (Supplementary Tables S3 and S8). These results showed that FTSH4 pulls down SLP1 and *vice versa*, strongly suggesting their specific interaction *in vivo*. We could not conduct a similar experiment for SLP2 due to a lack of specific antibodies.

### Two distinct types of complexes containing FTSH4 are present in *Arabidopsis* mitochondria: a megacomplex with SLP1 and SLP1-free smaller complexes

**Figure 1:**
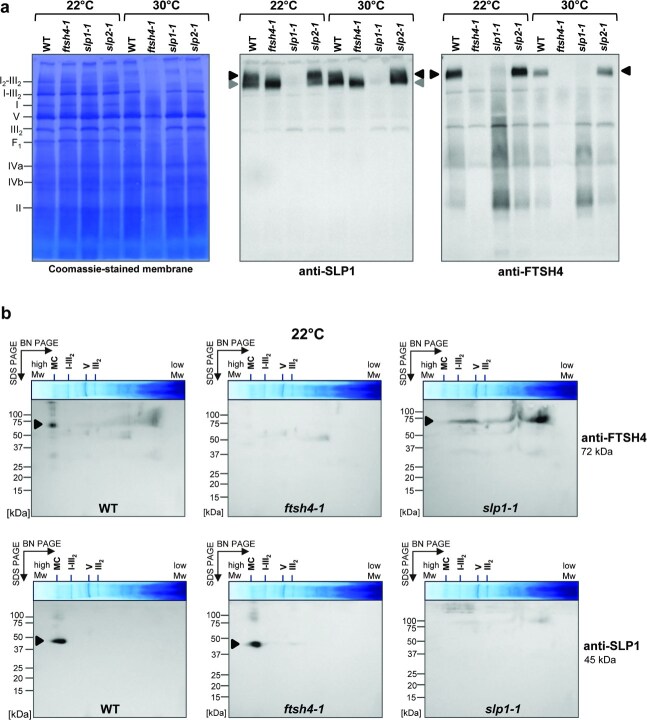
BN-PAGE and 2D BN/SDS–PAGE analysis of FTSH4 and SLP1 complexes in *Arabidopsis* mitochondria. (a) FTSH4 and SLP1 complexes identified by immunoblotting after BN-PAGE. Mitochondria from 2-week-old WT, *ftsh4-1*, *slp1-1*, and *slp2-1* plants grown under optimal conditions (22°C) or moderate heat stress (30°C) were solubilized with digitonin (5 g/1 g), and proteins were separated by 3%–12% BN-PAGE followed by immunodetection of FTSH4 and SLP1. The representative Coomassie-stained membrane and immunoblots are shown. Black arrowheads—FTSH4–SLP1 megacomplex; gray arrowheads—FTSH4-free, SLP1-containing complex. The OXPHOS complexes indicated on the left served as molecular weight markers. Their molecular weights were assigned according to the work of Eubel et al. (2003) and Braun (2020) as follows: I_2_–III_2_: 2500 kDa; I–III_2_: 1500 kDa; I: 1000 kDa; V: 650 kDa; III_2_: 500 kDa; F1: 350 kDa; IVa: 300 kDa; IVb: 220 kDa; II: 160 kDa. (b) Monomeric FTSH4 and SLP1 proteins identified by immunoblotting after BN/SDS-PAGE. Mitochondrial proteins from 2-week-old WT, *ftsh4-1*, and *slp1-1* plants grown at 22°C were separated by BN-PAGE as described above, and the second-dimension electrophoresis was run in a two-step tricine-SDS gel (8% and 10%) followed by immunodetection of FTSH4 and SLP1. Representative immunoblots with the corresponding BN-PAGE gel lanes are shown. The positions of several OXPHOS complexes as well as the FTSH4–SLP1 megacomplex (MC) are indicated at the top of the BN-PAGE gel lane, and the sizes of the molecular weight markers are on the left side of the immunoblots. Black arrowheads mark the position of monomeric forms of FTSH4 (top panel) and SLP1 (bottom panel).

To investigate the putative complex containing SLP1 and FTSH4, whose existence was indicated by the above results, blue-native polyacrylamide gel electrophoresis (BN-PAGE) followed by Western blotting was performed for mitochondria isolated from the WT, *ftsh4-1*, *slp1-1*, and *slp2-1* plants grown at 22°C or 30°C. At both temperatures, two closely localized complexes detected with an anti-SLP1 antibody were observed in WT mitochondria but not in *slp1-1* ones (Fig. 1a). The lower band migrated near the position of the I_2_III_2_ supercomplex, the size of which is estimated at ~2.5 MDa (Fig. 1a, gray arrow) (Eubel et al. 2003, Braun 2020). This SLP1 complex was not recognized by an anti-FTSH4 antibody, implying that it contains SLP1 but not FTSH4. In contrast, the higher-located SLP1-containing complex was also recognized by the anti-FTSH4 antibody; it had an estimated mass of ~3–4 MDa and will be further referred to as “megacomplex” (Fig. 1a, black arrowhead). The megacomplex was absent in both the *ftsh4-1* and *slp1-1* knockout mutants, which confirmed that it contained the FTSH4 and SLP1 proteins. This was verified further by visualizing monomeric FTSH4 and SLP1 as spots with expected molecular weights of 72 and 45 kDa, respectively, by two dimensional blue-native/sodium dodecyl sulfate polyacrylamide gel electrophoresis (2D BN/SDS-PAGE) combined with western blotting (Fig. 1b and Supplementary Fig. S1) of WT mitochondrial proteins. Notably, the 2D BN/SDS-PAGE analysis did not show the presence of rhomboid-like protein 12 (RBL12), a homolog of the human rhomboid protease PARL (Kmiec-Wisniewska et al. 2008), in the FTSH4- and SLP1-containing megacomplex (Supplementary Fig. S2).

At both temperatures tested (22°C and 30°C), two SLP1 complexes, with and without FTSH4, were present in WT and *slp2-1* mitochondria, albeit at different ratios (Fig. 1a). Under the optimal growth conditions (22°C), the ~3–4 MDa FTSH4–SLP1 megacomplex was slightly more abundant, whereas, at the moderately elevated temperature, the complex lacking FTSH4 became dominant. The noticeably lower abundance of the FTSH4–SLP1 megacomplex at the elevated temperature was unexpected, given the known protective role of FTSH4 against heat stress (Maziak et al. 2021).

A detailed analysis of the immunoblots decorated with the anti-FTSH4 antibody revealed an unexpected feature: in all the lines except for *ftsh4,* FTSH4 was detected not only as part of the megacomplex but also in diverse assemblies of considerably lower molecular weight. These assemblies migrated as fuzzy wide bands in BN-PAGE, the smallest one of ~160 kDa giving the strongest signal (Fig. 1a), and as a smear from ~160 kDa to 1.5 MDa in 2D BN/SDS-PAGE (Fig. 1b and Supplementary Fig. S1). Since no signal was present in those regions when the blots were probed with an anti-SLP1 antibody, we named these entities as SLP1-free FTSH4 LMW complexes. In WT and *slp2-1* mitochondria, the FTSH4–SLP1 ~3–4 MDa megacomplexes were the predominant form, while the SLP1-free LMW complexes were the only form of FTSH4 complexes in *slp1-1*, and their amount was much higher than in WT and *slp2-1* (Fig. 1 and Supplementary Fig. S1).

To investigate the nature of the FTSH4 complexes, we studied them in the *ftsh4-3* mutant line in which FTSH4 is truncated and lacks the plant-specific, alanine-rich C-terminus fragment of FTSH4 (Urantowka et al. 2005, Gibala et al. 2009). This truncation did not affect the formation of the megacomplex or the LMW complexes, although the latter showed higher electrophoretic mobility than the WT ones, which reflected the decreased mass of the truncated FTSH4 (Supplementary Fig. S3). Thus, the C-terminus of FTSH4 seems not to take part in the assembly of the complexes in question.

**Figure 2:**
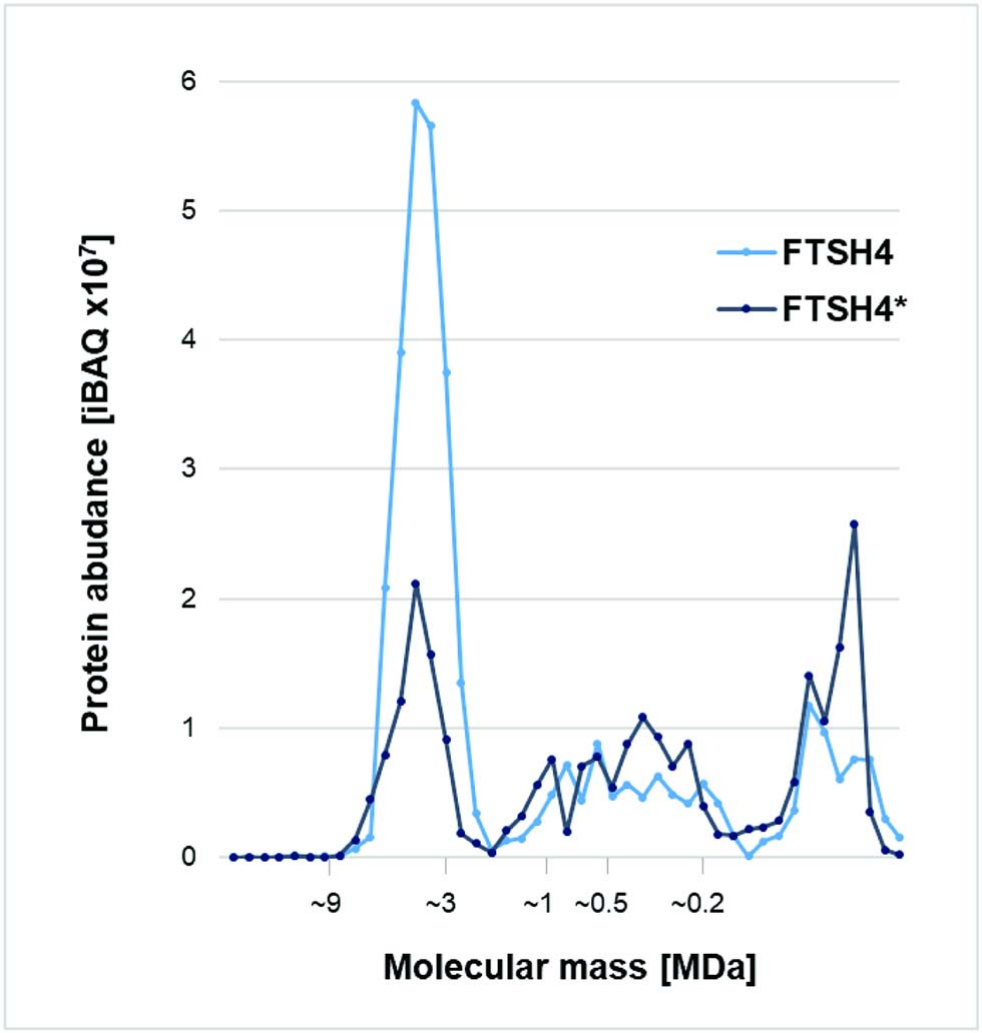
Distribution of FTSH4 in diverse protein complexes separated by large-pore (lp) BN-PAGE. Analysis of data published by Rugen et al. (2019) for cross-linked (FTSH4) and non-cross-linked (FTSH4^*^) WT mitochondria. A comparison of migration profiles of FTSH4: the gel was cut into 48 slices and FTSH4 was quantitated in each slice by mass spectrometry. The protein abundance is based on iBAQ values (intensity-based absolute quantification) without normalization.

To complement the study of the FTSH4 status in *Arabidopsis* mitochondria, we analyzed the complexome profiling data of Rugen et al. (2019) based on large-pore (lp) BN-PAGE in conjunction with mass spectrometry. The migratory profile of FTSH4, with or without cross-linking, is consistent with our earlier finding that it can be present in two forms: as an ~3–4 MDa megacomplex and as smaller assemblies ranging from ~200 kDa to ~1.5 MDa (Fig. 2), corresponding to the smear observed in 2D BN/SDS-PAGE (Fig. 1b and Supplementary Fig. S1). A comparison of the FTSH4 profile obtained for cross-linked and non-cross-linked mitochondria showed a significantly lower relative abundance of the FTSH4 megacomplex in the non-cross-linked samples with only a slightly higher abundance of the smaller assemblies (from ~200 kDa to ~1.5 MDa) and an additional pronounced signal at ~100 kDa (Fig. 2). Considering the proportion between the cross-linked and non-crossed samples, it is reasonable to assume that the assemblies with a mass of 200 kDa to ~1.5 MDa are rather stable forms, whereas the 100-kDa form is derived from the disassembly of the FTSH4–SLP1 megacomplex.

Next, we subjected the Rugen et al. (2019) data to hierarchical clustering to group the proteins according to their migration profiles to predict likely components of cross-linked FTSH4 complexes (Supplementary Fig. S4a). Contrary to our expectations, SLP1 did not group with FTSH4. A comparison of the migration profiles of FTSH4, SLP1, and SLP2 provides a visual explanation of this result (Supplementary Fig. S4b and c). The peak representing SLP1 has an asymmetric shape, likely reflecting the presence of only a small fraction of SLP1 in assemblies with FTSH4 overlapped by a prominent peak formed by the majority of SLP1 in complex with the SLP2 protein.

Among the proteins comigrating with FTSH4 in the megacomplex (Supplementary Fig. S4a), our attention was drawn to a plant-specific protein of unknown function encoded by the *AT3G51100* gene. This protein was also identified in FTSH4 immunoprecipitates at both 22°C and 30°C (Supplementary Tables S1, S2, S6, and S7). Previously, using iTRAQ-based proteomics, we found the AT3G51100 protein accumulating in mitochondria of *ftsh4-1* plants grown at 22°C or 30°C (Maziak et al. 2021). These results suggest that this plant-specific protein may be a subunit of the megacomplex. However, it was not identified in reciprocal coimmunoprecipitation using anti-SLP1 antibodies in the *qrt1-2* background. This may be due to the instability in its interaction with the megacomplex or due to technical limitations such as low abundance or inaccessible epitopes. Without further experimental validation, the role of AT3G51100 as a subunit of the FTSH4–SLP1 megacomplex remains speculative.

### The membrane topology of FTSH4 is the same in the megacomplex and the SLP1-free smaller complexes

**Figure 3:**
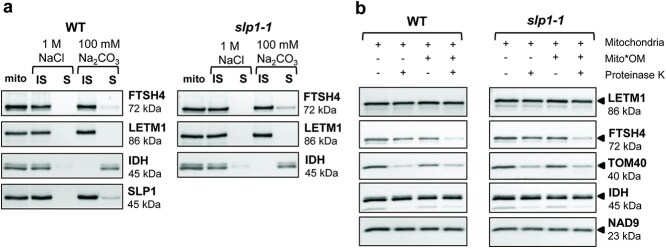
Submitochondrial localization and topology of FTSH4. (a) Mitochondria isolated from WT and *slp1-1* plants grown at 22°C were treated with 1 M sodium chloride (NaCl) or 100 mM sodium carbonate (Na_2_CO_3_) to fractionate proteins into soluble and membrane-bound fractions. After centrifugation, supernatant and pellet proteins were separated by SDS-PAGE and detected by immunoblotting. FTSH4, LETM1, and SLP1 were used as markers of the membrane-bound protein fractions and IDH as a soluble protein marker. Mito, untreated mitochondria; IS, insoluble (membrane) proteins; S, soluble proteins. (b) Mitochondria were subjected or not to osmotic shock to rupture the outer mitochondrial (OM) membrane and the intact mitochondria and mitoplasts were treated with proteinase K to determine the topology of selected proteins. Proteins were separated by SDS-PAGE and detected by immunoblotting. TOM40 is an integral OM protein, FTSH4 and LETM1 are inner membrane (IM) proteins with soluble regions exposed to the intermembrane space (FTSH4) or matrix (LETM1), NAD9 is an integral IM protein, and IDH is a soluble matrix protein. The anti-FTSH4 antibody recognizes the N-terminal region of FTSH4. Mito^*^OM, mitoplasts. Representative immunoblots are shown.

Until now, the FTSH4 protease was believed to exist solely as a megacomplex anchored in the inner mitochondrial membrane, with the catalytic part directed toward the IMS (Urantowka et al. 2005). However, the present findings indicate that FTSH4 can also form smaller assemblies lacking SLP1, which do not result from the disassembly of the megacomplex. Given that the *slp1-1* mutant contains only LMW complexes of FTSH4, we decided to investigate the location and topology of the FTSH4 protease in *slp1-1* mitochondria, i.e. in the small complexes. Two approaches were used: separation of soluble and membrane proteins after treating isolated mitochondria with sodium carbonate and treating mitoplasts with proteinase K. As shown in Fig. 3a, FTSH4 was found mainly in the membrane fraction of both *slp1-1* and WT mitochondria and showed similar sensitivity to proteinase K treatment in *slp1-1* and WT mitoplasts (Fig. 3b). Collectively, these results indicate that FTSH4 is an integral part of the inner membrane and exposes its catalytic domain to the IMS.

### The FTSH4 protein level increases considerably in the absence of SLP1

**Figure 4:**
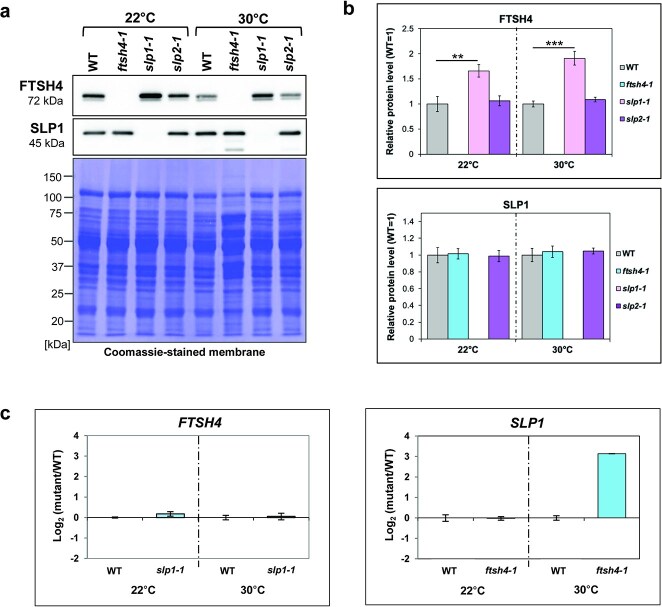
Protein and transcript levels of FTSH4 and SLP1. (a) Mitochondrial proteins isolated from 2-week-old WT, *ftsh4-1*, *slp1-1*, and *slp2-1* plants grown at 22°C and 30°C were separated by SDS-PAGE and FTSH4 and SLP1 proteins were detected by immunoblotting. Representative immunoblots and Coomassie-stained membranes are shown. (b) FTSH4 and SLP1 were quantified densitometrically from the immunoblots using ImageJ (Fiji) software with a Coomassie-stained membrane as the loading control. The protein abundance is given relative to that in the WT. Data are mean ± SD of three independent biological replicates. ^**^P ≤ 0.01; ^***^P ≤ 0.001. (c) The levels of the *FTSH4* and *SLP1* transcripts in WT, *ftsh4-1*, and *slp1-1* plants were determined by RT-qPCR and are expressed as a log_2_ ratio (mutant/WT). The values are the mean of a minimum of three independent biological replicates, with the error bars representing the SD.

The absence of the FTSH4–SLP1 megacomplex and the increased abundance of SLP1-free LMW FTSH4 complexes in the *slp1-1* mutant prompted us to investigate how the lack of SLP1 affects the expression of FTSH4. Figure 4a and b shows that the FTSH4 protein level is substantially elevated in *slp1-1* mitochondria (almost double) but not in *slp2-1* ones relative to the WT at both 22°C and 30°C. In contrast, the *FTSH4* transcript level is the same in *slp1-1* and the WT (Fig. 4c), indicating that the increased FTSH4 protein abundance in *slp1-1* is due to posttranscriptional regulation. In turn, the SLP1 protein level is the same in the *ftsh4* mutant and the WT at both growth temperatures (Fig. 4a and b), while the *SLP1* transcript level is significantly more abundant in the *ftsh4-1* mutant at 30°C but similar to that in the WT at 22°C (Fig. 4c). These results indicate a complex relationship between the transcript and protein levels of FTSH4 and SLP1.

### Morphological abnormalities of *slp1-1* plants at 30°C are less pronounced compared with those of *ftsh4* plants and concern meristematic tissues

**Figure 5:**
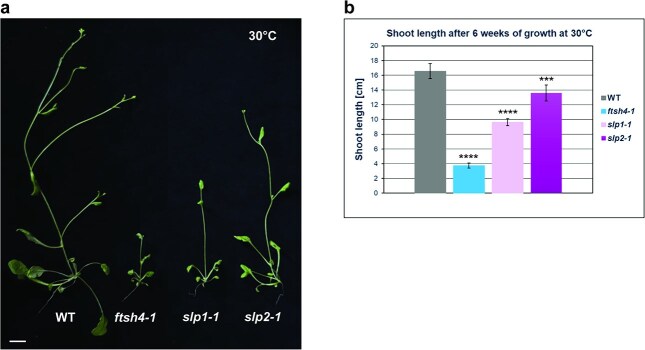
Morphology of WT, *ftsh4-1*, *slp1-1*, and *slp2-1* plants. (a) Six-week-old plants grown in soil at 30°C. The scale bar indicates 1 cm. (b) Length of shoots of 6-week-old plants grown in soil at 30°C. The data represent mean ± SD. ^***^P ≤ 0.0001; ^****^P ≤ 0.00001 (*n* = 10 for WT and *slp2-1*; *n* = 3 for *ftsh4-1*, *n* = 4 for *slp1-1*).

To assess the functionality of the SLP1-free, LMW FTSH4 complexes, we compared the phenotype of the *slp1-1* mutant, in which FTSH4 is present only in the form of the LMW complexes, with that of the *ftsh4-1* mutant, in which FTSH4 is absent, focusing on traits specific to *ftsh4-1.* One characteristic of the *ftsh4-1* mutant is an altered plant morphology at a moderately elevated temperature (30°C) but not under optimal growth conditions (22°C). Similarly to *ftsh4-1*, the *slp1-1* mutant was morphologically normal at 22°C (Supplementary Figs. S5a and S6a). However, when plants were grown at 30°C either on agar plates or in soil, the *slp1-1* mutant exhibited reduced rosette size and shorter root and shoot lengths compared to WT plants, although these aberrations were less pronounced than those of the *ftsh4-1* mutant (Fig. 5a and b and Supplementary Figs. S5b and e and S6b). The reduction of shoot length and rosette size of *slp1-1* plants was observed throughout the studied growth period and was ~50% of that observed for WT plants at the age of 6 weeks (Fig. 5 and Supplementary Fig. S5e). Furthermore, an analysis of plant growth over a period of 6 weeks revealed a developmental delay in the appearance of true leaves in the *slp1-1* mutant, similar to that observed in the *ftsh4-1* mutant (Smakowska et al. 2016). This resulted in a lower total leaf number in the mutant compared to WT plants of the same chronological age (Supplementary Fig. S5d). A developmental delay of *slp1-1* was also observed in the emergence of a flower stalk, with the *slp1-1* inflorescence stems being significantly shorter and immature at the age of 6 weeks compared to WT plants, suggesting a delay in the onset of flowering (Supplementary Fig. S5c). These developmental abnormalities resemble those of *ftsh4-1* mutants subjected to mild heat stress (Dolzblasz et al. 2016, 2018, Smakowska et al. 2016), where defects in root and shoot growth, as well as inflorescence formation, have been linked to the premature cessation of the root and shoot apical meristems (SAMs). The pronounced reduction in shoot and inflorescence growth observed in *slp1-1*, therefore, likely reflects the specific role of the FTSH4–SLP1 megacomplex in maintaining SAM activity and developmental progression under temperature stress. Notably, no such phenotypic alterations were observed for *slp2-1* plants at either temperature (Fig. 5 and Supplementary Figs. S5c–e and S6).

### The SLP1-free small FTSH4 complexes show a proteolytic and a chaperone-like activity

**Figure 6:**
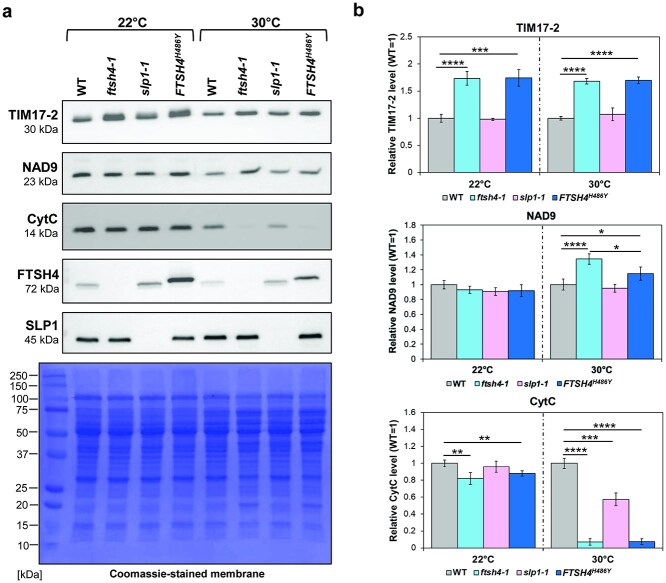
Abundance of TIM17-2, NAD9, and CytC. (a) Mitochondrial proteins isolated from 2-week-old WT, *ftsh4-1*, *slp1-1*, and *FTSH4*^*H486Y*^ plants grown at 22°C and 30°C were separated by SDS-PAGE, and FTSH4 substrates were visualized by immunoblotting. Representative immunoblots and a Coomassie-stained membrane are shown. FTSH4 and SLP1 were used as control. (b) TIM17-2, NAD9, and CytC were quantified densitometrically from the immunoblots using ImageJ (Fiji) software with a Coomassie-stained membrane as a loading control. The protein abundance is given relative to that in the WT. Data are mean ± SD of a minimum of four independent biological replicates. ^*^P ≤ 0.05; ^**^P ≤ 0.01; ^***^P ≤ 0.001; ^****^P ≤ 0.0001.

The relatively mild morphological defects of the *slp1-1* plants vis-à-vis the *ftsh4-1* ones documented above suggest that FTSH4 is at least partially functional even in the absence of SLP1. We verified this assumption directly by studying the activity of FTSH4 toward its known substrates in *slp1-1* mitochondria. The best-known proteolytic substrate of FTSH4 is the TIM17-2 protein (Opalinska et al. 2018). It accumulates to the same level when the FTSH4 protease is absent (*ftsh4-1*) and when its proteolytic activity is abolished (*FTSH4*^*H486Y*^), regardless of the growth conditions (Fig. 6a and b). The *slp1–1* defect had no effect on the TIM17–2 abundance since it was similar to that in WT (Fig. 6a and b). Another well-known substrate of FTSH4 is NAD9, a subunit of respiratory chain complex I (Maziak et al. 2021). Unlike TIM17-2, NAD9 is targeted by FTSH4 exclusively under thermal stress and its degradation is apparently due to both functions of FTSH4—the proteolytic one and the chaperone one. This latter conclusion can be drawn from the observation that the NAD9 accumulation is greater at 30°C in the *ftsh4-1* line than in the *FTSH4*^*H486Y*^ one (Maziak et al. 2021; Fig. 6a and b). As found earlier for TIM17-2, the NAD9 level also was not affected by the lack of SLP1 (Fig. 6a and b). It is important to note, however, that the steady-state level of FTSH4 in *slp1-1* is nearly double that in the WT (Fig. 4a and b), which suggests that the specific activity of FTSH4 in the SLP1-free small complexes is in fact lower than the activity of the megacomplex.

Our earlier findings showed a highly reduced level of cytochrome c (CytC) in the mitochondria of *ftsh4-1* plants grown at 30°C (Maziak et al. 2021). Here, we showed that this reduction depends mainly on the proteolytic activity of FTSH4 as the proteolytically inactive version of FTSH4 (*FTSH4*^*H486Y*^) caused a nearly identical effect to that in *ftsh4-1* (Fig. 6a and b). To reiterate, the absence of proteolytic activity of FTSH4 paradoxically leads to a loss of the CytC protein. This indicates that the CytC protein is not a substrate of FTSH4 itself, but rather its level is indirectly influenced by the proteolytic activity of FTSH4. Also, in the *slp1-1* plants grown at 30°C, the level of CytC was decreased relative to that in WT mitochondria, but this decrease was less profound than in *ftsh4-1* or *FTSH4*^*H486Y*^ (Fig. 6a and b). This result combined with the earlier ones indicates that it is predominantly the proteolytic activity of the FTSH4 megacomplex that is required for CytC stability at 30°C and that it can only partially be replaced by the activity of the SLP1-free LMW complexes of FTSH4.

**Figure 7:**
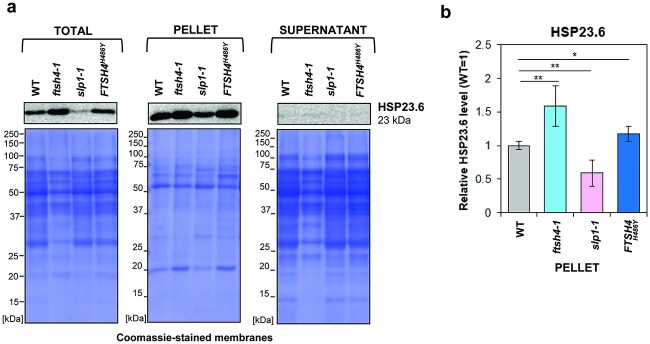
Abundance of HSP23.6 aggregates in mitochondria of plants subjected to moderate heat stress. (a) Mitochondrial proteins isolated from WT*, ftsh4-1*, *slp1-1*, and *FTSH4*^*H486Y*^ plants grown at 30°C were treated with Triton X-100 and separated by SDS-PAGE, and the HSP23.6 protein abundance was assessed by immunoblotting. One-third of the isolated mitochondrial material was analyzed as the TOTAL fraction, while the remaining two-thirds were subjected to detergent extraction and fractionated into PELLET and SUPERNATANT. Representative immunoblots and Coomassie-stained membranes are shown. TOTAL, total mitochondrial protein fraction; PELLET, Triton X-100-insoluble protein aggregates; SUPERNATANT, soluble proteins. (b) HSP23.6 abundance in the pellet (the intensity of immunological signal) was quantified densitometrically using ImageQuant. Abundance values are given relative to the value obtained for the WT set to 1. The protein abundance is given relative to that in the WT. Data are mean ± SD of a minimum of three independent biological replicates. ^*^P ≤ 0.05; ^**^P ≤ 0.01.

We have previously reported that both the proteolytic and chaperone-like activities of FTSH4 are required to protect mitochondrial proteins against aggregation induced by long-term stress at the moderate temperature of 30°C (Maziak et al. 2021). Small heat shock proteins (e.g. HSP23.6) comprise most of the insoluble aggregates of mitochondrial proteins formed under such conditions (Maziak et al. 2021). In the current study, we investigated the accumulation of HSP23.6 in the Triton X-100–insoluble mitochondrial fraction (pellet fraction) isolated from plants grown at 30°C. As expected, the *ftsh4-1* mitochondria contained more insoluble HSP23.6 than the WT mitochondria, while the *slp1-1* mutant showed, rather unexpectedly, the lowest content of aggregated HSP23.6, even lower than in the WT (Fig. 7).

**Figure 8:**
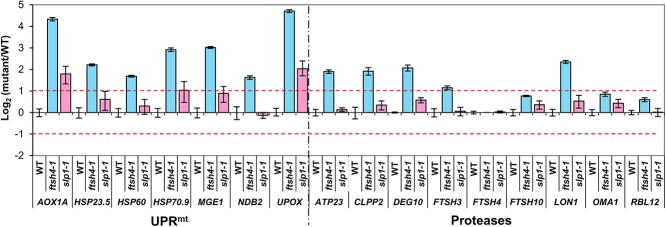
Transcript levels of selected marker genes of the mitochondrial unfolded protein response (UPR^mt^) and mitochondrial proteases in plants grown under moderate heat stress. The level of transcripts in 2-week-old *ftsh4-1* and *slp1-1* plants grown at 30°C was determined by RT-qPCR and expressed as a log_2_ ratio (mutant/WT). Values < −1 and >+1, indicating an over two-fold down- or upregulation in the mutant, are denoted by dashed lines. The data shown are mean ± SD from at least three biological replicates.

In line with the above results highlighting a low propensity of proteins for aggregation in *slp1-1* mitochondria under moderate heat stress, the upregulation of the markers associated with mitochondrial unfolded protein response (UPR^mt^) (Tran and van Aken 2020) observed in the *ftsh4-1* mutant compared to the WT was significantly less pronounced in the *slp1-1* mutant (Fig. 8). Only the *AOX1A* and *UPOX* transcripts showed some accumulation under moderate heat stress, suggesting minor oxidative stress in the *slp1-1* mitochondria (Fig. 8).

### The mitochondrial protein quality control system is almost unaltered at the transcript level in the *slp1-1* mutant

The low level of protein aggregates formed in the *slp1-1* mitochondria following prolonged exposure to a moderately high temperature prompted us to ask whether this was not caused by an overexpression of the mitochondrial protein quality control (mtPQC) system comprising, among others, diverse proteases and chaperones. The transcript levels of several mitochondrial chaperones (HSP60-2, mtHSC70-1, HSP23.5, and MGE1) were similar in *slp1-1* and WT plants grown at 30°C (Fig. 8). Furthermore, none of the ATP-dependent proteases, components of the plant mtPQC system (FTSH4, FTSH3, FTSH10, LON1, and CLPP2) were upregulated at the transcript level in *slp1-1* (Fig. 8). Similarly, the transcript levels of several mitochondrial ATP-independent proteases (OMA1, DEG10, ATP23, and RBL12), located, similarly to FTSH4, in the IM or IMS, were unaltered in the *slp1-1* mutant mitochondria (Fig. 8).

## Discussion

We showed here that in *Arabidopsis* mitochondria, the *i*-AAA protease FTSH4 and the stomatin-like protein SLP1 form a membrane-anchored megacomplex similar to that formed by the respective orthologs in humans (YME1L and SLP2; Wai et al. 2016) and in the protozoan parasite *Trypanosoma* (TbYme1 and TbSlp2; Serricchio and Bütikofer 2021). The size, composition, and function of these megacomplexes, however, appear to be organism-specific. Recently, two independent cryoelectron microscopy studies by Qiao et al. (2022) and Ma et al. (2022) revealed the high-resolution structure of the FtsH/HflK/HflC complex in *Escherichia coli* cell membranes. Four FtsH hexamers and 12 copies of both HflK and HflC proteins belonging to the SPFH (stomatin, prohibitin, flotillin, and HflK/C) family form a large, cage-like complex of 2.7 MDa (Ma et al. 2022).

The *i*-AAA-stomatin megacomplex identified in *Arabidopsis* is the largest known to date, its molecular mass of ~ 3–4 MDa substantially exceeding those of the human and *Trypanosoma* megacomplexes (~2 and ~1.5 MDa, respectively) (Wai et al. 2016, Serricchio and Bütikofer 2021). It should be emphasized that the *i*-AAA protease cannot form a megamolecular complex in the absence of the respective stomatin [SLP1 in *Arabidopsis* (Fig. 1a) or SLP2 in humans and *Trypanosoma*], which indicates a conservative role of the stomatins in stabilizing/forming the *i*-AAA–stomatin megacomplexes. The exact composition and stoichiometry of the mitochondrial megacomplexes described to date are unknown, although there are indications that proteins other than an *i*-AAA protease and a stomatin are also involved. For instance, the human megacomplex contains another inner membrane protease, the rhomboid protease PARL (Wai et al. 2016). Unlike the human megacomplex, the plant mitochondrial rhomboid protease RBL12 appears not to be a part of the *Arabidopsis* megacomplex (Supplementary Fig. S2).

We found that in WT *Arabidopsis,* FTSH4 exists in two forms: the dominant one, a megacomplex that contains SLP1, and smaller assemblies without SLP1, the abundance of which is relatively low. In *slp1-1* mitochondria, only the LMW forms of FTSH4 are present, and their abundance is markedly increased despite unchanged transcript levels. This suggests posttranscriptional regulation of FTSH4, likely due to enhanced protein stability in the absence of SLP1. In contrast, in *ftsh4* mutants, *SLP1* transcripts increase significantly at elevated temperature (30°C), while protein level remains unchanged. This strong transcriptional upregulation is likely a compensatory response to reduced SLP1 protein stability under heat stress, when SLP1 cannot be incorporated into the FTSH4-containing megacomplex and is prone to degradation. Together, these findings reveal a mutual dependency between SLP1 and FTSH4, where each protein influences the stability and accumulation of the other.

Similar SLP1-free LMW assemblies of mitochondrial *i*-AAA proteases have been reported in other organisms. Human YME1L forms ~200-kDa complexes (Wai et al. 2016), while *Trypanosoma* TbYme1 is found in oligomeric structures ranging from 250 kDa to 1 MDa (Serricchio and Bütikofer 2021). Our analysis using BN-PAGE, 2D BN/SDS-PAGE, and the complexome profiling data of Rugen et al. (2019), with and without cross-linking, revealed that the FTSH4 assemblies lacking SLP1 range in mass from ~160–200 kDa to ~1.5 MDa. They were discovered in comparable levels in samples with and without cross-linking, suggesting that most of them exist in stable forms *in vivo*. Considering the molecular weights observed in native complexome analysis and the size of the FTSH4 monomer (~77 kDa), these assemblies may include dimers, trimers, hexamers, or higher-order structures composed of multiple hexamers. Alternatively, other proteins may be part of these complexes. However, we also cannot exclude that some assemblies represent forms generated during the assembly or disassembly of larger FTSH4 complexes. Such disassembly may occur naturally in mitochondria or be induced during sample preparation, particularly by detergent solubilization and electrophoresis. Taken together, the SLP1-free LMW assemblies of FTSH4 likely comprise a mixture of stable oligomers and transient species. Further structural studies will be required to resolve their precise composition and organization.

The key question addressed in this study is whether the LMW complexes of FTSH4, which lack SLP1, are functional and can substitute for the FTSH4–SLP1 megacomplex. To investigate this, we analyzed the levels of known FTSH4 substrates in *slp1-1* mitochondria, where FTSH4 is exclusively present in LMW assemblies. Despite the loss of SLP1 and, consequently, the FTSH4–SLP1 megacomplex, there was no accumulation of the FTSH4 proteolytic substrates TIM17-2 (Opalinska et al. 2018) and NAD9 (Maziak et al. 2021) in *slp1-1* mitochondria. Thus, the SLP1-free LMW assemblies of FTSH4 are functional as proteases—at least toward the two principal FTSH4 substrates. The hexameric organization of FTSH proteases is widely considered to be essential for full proteolytic activity. However, smaller oligomeric forms, such as trimers or dimers, may exhibit partial activity, albeit generally reduced compared with hexamers (Yoshioka and Yamamoto 2011). Therefore, it is plausible that the LMW complexes in *slp1-1* include a mixture of active oligomeric forms with varying efficiencies. Notably, the total level of FTSH4 protein in *slp1-1* mitochondria is significantly higher than in the WT (Fig. 4). Thus, the apparent preservation of proteolytic efficiency in *slp1-1* mitochondria may result from a nearly two-fold increase in FTSH4 protein abundance, which compensates for the lower specific activity of SLP1-free LMW FTSH4 complexes.

Surprisingly, another substrate of FTSH4, the HSP23.6 protein, behaves differently. A reduced level of HSP23.6 was noted in *slp1-1* mitochondria compared to the WT after prolonged moderate temperature stress (Fig. 7). We have previously shown that both the proteolytic and chaperone-like activities of FTSH4 are important in preventing the aggregation of mitochondrial proteins, including HSP23.6, at moderate heat stress (30°C) (Maziak et al. 2021). The lower abundance of the HSP23.6 aggregates in the *slp1-1* mitochondria leads us to speculate that the SLP1-free LMW FTSH4 assemblies are highly effective in preventing aggregation of thermally unfolded proteins. FTSH4 most likely prevents protein aggregation by binding to and stabilizing unfolded proteins and/or by degrading misfolded proteins. Unlike the straightforward proteolytic activity of FTSH4, this mode of action could be more effective if the enzyme were smaller and more mobile, thereby providing easier access to the substrate. Compared with the FTSH4–SLP1 megacomplex, the LMW complexes of FTSH4 more closely meet this criterion.

The morphological comparison of *ftsh4-1*, *slp1-1*, and WT plants at 30°C (Fig. 5 and Supplementary Figs. S5 and S6) suggests that the elevated levels of SLP1-free LMW complexes of FTSH4 in *slp1-1* plants do not fully compensate for the absence of the FTSH4–SLP1 megacomplex, which predominates in WT mitochondria. Several phenotypic abnormalities previously associated with the loss of FTSH4 under heat stress, such as reduced leaf number and rosette size, shorter inflorescence stems, and delayed or absent flowering (Smakowska et al. 2016), have also been observed in *slp1-1* plants, although in a milder form. These phenotypes could be explained by a dysfunction of meristematic tissues, although direct confirmation is still required. Our previous studies (Dolzblasz et al. 2016, 2018) have demonstrated that the reduced inflorescence stem length in *ftsh4* mutants results from the premature arrest of the SAM. These findings emphasize the essential role of FTSH4 in maintaining mitochondrial function within the SAM, which is critical for sustaining stem cell activity during development. Our current findings refine this model by identifying SLP1 as a partner of FTSH4 within a megacomplex. This suggests that the integrity and function of the SAM depend not only on FTSH4 alone but also on its coordinated action with SLP1. The presence of SLP1-free FTSH4 complexes in *slp1-1* mutants is insufficient to maintain full meristematic activity under elevated temperatures, indicating that the FTSH4–SLP1 megacomplex is functionally superior in supporting mitochondrial performance in these tissues.

SLP1 likely serves as a scaffold protein that facilitates the assembly and stabilization of mitochondrial respiratory supercomplexes, particularly those involving complex I (Gehl et al. 2014). This function appears to be mediated through interactions with specific lipids such as cardiolipin, which organize membrane microdomains and facilitate the integration of protein complexes into the mitochondrial membrane (Gehl and Sweetlove 2014). In turn, FTSH4 regulates cardiolipin abundance and contributes to the assembly/stability of complexes I and V (Kolodziejczak et al. 2007, Smakowska et al. 2016). Together, these findings strongly support a cooperative role of SLP1 and FTSH4 in shaping mitochondrial membrane architecture via cardiolipin, thereby enhancing respiratory efficiency—a process particularly critical during energy-demanding developmental stages or under stress. Consistent with this, the *slp1-1/ftsh4-1* double mutant displays synthetic lethality and fails to germinate (unpublished results), highlighting the indispensable and nonredundant function of the FTSH4–SLP1 megacomplex. Overall, our phenotypic data indicate that this megacomplex plays a unique and essential role that cannot be compensated for by SLP1-free FTSH4 LMW assemblies and that it is crucial for sustaining meristem activity during critical developmental checkpoints under stress.

## Materials and Methods

### Plant materials and growth conditions

*Arabidopsis thaliana* WT and transgenic lines *ftsh4-1* (SALK_035107), *FTSH4*^*H486Y*^, and *ftsh4-3* (SAIL_1283_F10) were in the Columbia-0 (Col-0) background. The *ftsh4-1* and *ftsh4-3* lines have been characterized by Gibala et al. (2009). The proteolytically inactive version of FTSH4 protease (*FTSH4*^*H486Y*^) was originally described by Opalinska et al. (2018). The T-DNA insertion mutants *slp1-1* (SAIL_210_D11) and *slp1-2* (SAIL_65_C05) were created in the *qrt1-2* mutant background (Copenhaver et al. 1998), while *slp2-1* (SALK_138526) was of the Col-0 ecotype. The *slp* lines were previously characterized by Gehl et al. (2014). The double *slp1-1/ftsh4-1* mutant was obtained by crossing the single *slp1-1* and *ftsh4-1* mutants. Plants were grown in a growth chamber on agar plates, in soil, or in liquid cultures at 22°C or 30°C under a long-day photoperiod (LD, 16 h light and 8 h dark) with a light intensity of 120 μmol m^−2^ s^−1^ and 60%–70% humidity. The appearance of the rosette leaves of plants grown in soil at 30°C was observed over a period of 6 weeks. After 6 weeks of growth in soil at 30°C, shoot length and rosette size measurements were conducted, and photographs of inflorescence stems were taken. For growth on agar plates, sterile seeds were plated on half-strength Murashige and Skoog (1/2 MS) medium supplemented with 1.5% sucrose and 1% bactoagar. For growth in liquid cultures, sterile seeds were added to a growth medium containing 1/2 MS, 3% sucrose, 2 mM 2-(N-morpholino)ethanesulfonic acid (MES), and 1.5 g/L Gamborg’s B-5 basal salt mixture, pH 5.7, and incubated on an orbital shaker at 80 rpm (Migdal et al. 2017).

### Isolation of mitochondria

Mitochondria were isolated according to the procedure described by Lyu et al. (2018) from 2-week-old plants grown in liquid culture at 22°C or 30°C.

### SDS-PAGE, BN-PAGE, and immunoblotting analyses

SDS-PAGE was performed according to Laemmli (1970), and 1D blue-native polyacrylamide gel electrophoresis (1D BN-PAGE) according to Schikowsky et al. (2018). Briefly, 50 μg of mitochondrial proteins was suspended in a 5 μL digitonin-containing buffer (30 mM HEPES, pH 7.4, 150 mM potassium acetate, 10% glycerol, 5% digitonin) and resolved by BN-PAGE in a 3%–12% Bis-Tris gel (Thermo Fisher Scientific) at 4°C. Two-dimensional BN/SDS-PAGE was carried out as described by Schägger (2001) using two-step (8% and 10%) tricine-SDS gels. After electrophoresis, proteins were blotted onto a polyvinylidene difluoride (PVDF) membrane (Bio-Rad) and probed with appropriate primary antibodies (Supplementary Table S4). The bound antibodies were visualized using appropriate horseradish peroxidase (HRP)-conjugated secondary antibodies and chemiluminescence with Clarity Western ECL (Bio-Rad) or Western Bright Quantum (Advansta) substrates and a GBox imager (Syngene). Band intensities were quantitated using Fiji ImageJ software (Fiji).

### Protein aggregation assay

The isolation of Triton X-100-insoluble protein aggregates from mitochondrial fractions was carried out as described in Maziak et al. (2021).

### Assessing the mitochondrial topology of FTSH4 protease

Fractionation of mitochondria into soluble and membrane-bound protein fractions using carbonate extraction and the proteinase K protection assay of intact mitochondria and mitochondria subjected to osmotic shock (mitoplasts) were carried out as described in Schäfer et al. (2022).

### Reverse transcription-quantitative PCR (RT-qPCR)

Total RNA was extracted from 2-week-old seedlings grown in liquid culture at 22°C or 30°C using the Gene Matrix Universal RNA Purification Kit (EURx) and subjected to reverse transcription quantitative polymerase chain reaction (RT-qPCR) analysis as described in Maziak et al. (2021) with the *ACT2* gene (AT3G18780) used as a reference. The primers used are listed in Supplementary Table S5.

### Statistical analysis

At least three independent biological replicates of each experiment were performed, and the means and standard deviations (SDs) of the mean were calculated. The statistical significance of differences between the means was determined using a two-tailed unpaired Student’s *t*-test. *P*-values were categorized as follows: ^*^P ≤ 0.05, ^**^P ≤ 0.01, ^***^P ≤ 0.001, and ^****^P ≤ 0.0001.

### Analysis of complexome profiling data

The complexome profiling data from Rugen et al. (2019) were utilized. Protein abundance profiles based on iBAQ (intensity-based absolute quantification; Schwanhäusser et al. 2011) values, as well as hierarchical clustering, were constructed with the NOVA software (version 0.8.0; Giese et al. 2015). If not otherwise indicated, no normalization was performed.

### FLAG-based affinity purification of FTSH4-interacting proteins

#### Sample preparation

Mitochondria isolated from four independent biological replicates of *ftsh4-1* and *FTSH4*^*H486Y*^ plants, each grown at 22°C or 30°C, were suspended in digitonin solubilization buffer (1% digitonin, 20 mM Tris–HCl, 0.1 mM EDTA, 100 mM NaCl, 10% glycerol, pH 7.7) at 1 mg protein/mL. Samples were supplemented with a phenylmethylsulfonyl fluoride (PMSF) and ethylenediaminetetraacetic acid (EDTA)-free protease inhibitor cocktail and incubated for 30 min at 4°C with end-over-end rotation. After centrifugation (18 000 × *g*, 10 min, 4°C), the solubilized material was loaded on an anti-FLAG affinity gel and incubated under constant rotation for 90 min at 4°C. After washing steps, proteins were eluted with Laemmli sample buffer. The purification procedure was verified by immunoblotting (Supplementary Fig. S7). In the next step, all samples were prepared using paramagnetic bead technology based on Single-Pot Solid-Phase-Enhanced Sample Preparation (SP3) (Hughes et al. 2019). Two types of SpeedBeads mixed in a ratio of 1:1 were used: GE45152105050250 and GE65152105050250 (Sigma-Aldrich). Proteins were reduced with dithiothreitol and alkylated with iodoacetamide. After that, the paramagnetic beads were added, the mixture was acidified with formic acid (FA), and protein binding to the beads was done by adding acetonitrile (ACN) to the final concentration of 50%. Beads with bound proteins were washed twice with 70% ethanol and twice with 100% ACN. Then, the proteins were digested with a trypsin/Lys-C mix (Promega) overnight. The resulting peptides were washed two times with 100% ACN and then eluted in four steps using (i) water, (ii) twice with1% dimethyl sulfoxide (DMSO), and (iii) with 2% DMSO. The collected peptides were dried and dissolved in a loading buffer (2% ACN with 0.05% trifluoroacetic acid) just before liquid chromatography-tandem mass spectrometry (LC-MS/MS) analysis.

#### LC-MS/MS analysis

LC-MS/MS was performed on a Q Exactive mass spectrometer (Thermo Fisher Scientific) coupled with a nanoHPLC (UltiMate 3000 RSLCnano System, Thermo Fisher Scientific) at the Proteomics and Mass Spectrometry Core Facility of the Malopolska Centre of Biotechnology (Jagiellonian University, Krakow, Poland). Peptides were loaded onto a trap column (AcclaimPep-Map100 C18, Thermo Fisher Scientific; ID 75 μm, length 20 mm, particle size 3 μm, pore size 100 Å) and then separated on a 50-cm analytical column (AcclaimPepMapRLSC C18, Thermo Fisher Scientific; ID 75 μm, particle size 2 μm, pore size 100 Å) in a 2-h gradient of acetonitrile (2%–40%) in the presence of 0.05% formic acid at a flow rate of 250 nL/min. The eluting peptides were ionized in a Digital PicoView 550 nanospray source (New Objective) and analyzed by the mass spectrometer in a data-dependent mode using the Top12 method. The MS and MS/MS spectra were acquired with a resolution of 70 000 and 17 500, respectively. The QCloud quality control system was used for monitoring the performance of the LC-MS/MS instrumentation during the measurements (Chiva et al. 2018, Olivella et al. 2021).

##### Data analysis

The LC-MS/MS data were processed with the Proteome Discoverer platform (v1.4; Thermo Fisher Scientific) and searched using an in-house MASCOT server (v2.5.1; Matrix Science) against the SwissTrEMBL *A. thaliana* database (release April 2022, 39 329 sequences) supplemented with the sequence of the mutant studied. The following parameters were applied—enzyme: trypsin; missed cleavages: up to 1; fixed modifications: carbamidomethyl (C); variable modifications: oxidation (M), acetyl (protein N-term); peptide mass tolerance: 10 ppm; and fragment mass tolerance: 20 mmu. The maximum false discovery rate (FDR) of peptide identification was set to 1%. Proteins were considered interactors when they met the following criteria: (i) they were identified in all the experiments in TEST (*FTSH4*^*H486Y*^) samples (4/4), (ii) they were identified with ≥2 peptides in at least one experiment in TEST samples, (iii) they were absent in CONTROL (*ftsh4-1*) samples plus proteins identified with two times higher average protein score in TEST samples in comparison to CONTROL, and (iv) they were identified with average score ≥ 40. The processed and analyzed data are presented in Supplemental Tables S6 and S7.

#### Statistical evaluation of selected interactors

The quality of the selected interactors was statistically evaluated by importing the data into Perseus (version 2.1.0.0; Tyanova et al. 2016). The analysis was performed using data that met the above selection criteria for interactors, except for criterion (iii). For proteins detected exclusively in the *FTSH4*^*H486Y*^ samples, missing values (“NaN”) were imputed with a constant value of “1” to enable subsequent statistical analysis and visualization. Prior to statistical analysis, the intensity values were log₂-transformed to approximate a normal distribution. A two-sided Student’s *t*-test with permutation-based false discovery rate (FDR) correction was performed to statistically compare the two experimental groups, controlling for multiple hypothesis testing (number of permutations = 250; FDR < 0.05; *S*₀ = 0.1). The results were visualized as a volcano plot, where log₂ fold change values were plotted against −log₁₀(*P*-values) (Supplementary Fig. S8). Proteins with a fold change ≥ 2 and a *P*-value ≤ 0.05 were considered to be statistically significant interactors. The tables presenting the statistically significant results can be found in Supplemental Tables S6 and S7 (volcano plot interactors). Proteins that met the FDR threshold were marked with an additional “+” in the results table. The volcano plot graph was created in Excel (Supplementary Fig. S8).

#### Protein–protein interaction by STRING database

Only proteins identified as statistically significant interactors in the Perseus analysis were analyzed using the STRING database (version 12.0; Szklarczyk et al. 2023) to explore potential functional relationships between proteins. The analysis was performed for *Arabidopsis thaliana* (organism code: 3702), using a minimum required interaction score of 0.4 (medium confidence). Known and predicted interactions based on experimental evidence, database annotations, gene co-occurrence, and text mining were included. The resulting protein–protein interaction (PPI) network was visualized in STRING and exported for graphical presentation (Supplementary Fig. S9).

### Protein cross-linking and coimmunoprecipitation of SLP1-interacting proteins

#### Sample preparation

Anti-SLP1 crude serum was clarified by centrifugation (10 000 × *g*, 10 min), glass wool filtration, and desalting against binding buffer (20 mM Na-phosphate buffer, pH 7.0). Rabbit immunoglobulins G (IgGs) were purified on protein G-Sepharose 4B fast flow (Sigma), essentially as described by the manufacturer. Purified IgGs were coupled to NHS-activated Sepharose 4 fast flow (GE Healthcare Life Sciences) as instructed by the manufacturer. Immunoprecipitation was carried out as described by Hajek et al. (2007). Briefly, 6 mg of mitochondrial proteins isolated from *qrt1-2* or *slp1-2* plants was cross-linked by incubation with 1% formaldehyde in 250 mM sucrose, 10 mM KCl, 0.15 mM MgCl_2_, and 20 mM HEPES, pH 7.2, for 30 min at 25°C, followed by quenching with 125 mM glycine. After centrifugation (18 000 × *g*, 10 min, 4 C), pellets were solubilized in 1% SDS, 1 mM EDTA, and 50 mM Tris-Cl, pH 7.5, for 30 min at 4 C followed by centrifugation, as described above. Solubilized proteins were diluted 20 times in buffer (0.5% Tween-20, 150 mM NaCl, 0.1 mM EDTA, and 50 mM Tris-Cl, pH 7.5) and incubated at 4°C overnight with anti-SLP1 IgGs cross-linked to Sepharose beads. The beads were spun down (2000 × *g*, 1 min) and washed five times in Tween buffer, followed by elution of bound proteins in 100 mM glycine and 500 mM NaCl, pH 2.8, and neutralization with Tris, pH 9.0. Eluted fractions were pooled and concentrated (Pierce Concentrators 9K) as instructed. Formaldehyde cross-linking was reversed by heating to 95°C for 30 min in 1× SDS loading buffer before separation on 10% SDS-PAGE mini gels. Gels were subjected to western blot analysis with anti-SLP1 antibodies (Supplementary Fig. S10a) or silver staining with the Pierce Color Silver Stain Kit (Supplementary Fig. S10b).

#### Protein identification

For protein identification, samples were run 1 cm into the separating gel, excised, and processed for identification at the Central Proteomics Facility, Dunn School of Pathology, University of Oxford (Oxford, UK). Gel pieces were destained in 25 mM ammonium bicarbonate in 50% acetonitrile, reduced (10 mM DTT, 37°C, 30 min) and alkylated (55 mM iodoacetamide, 25°C, 1 h), and digested with trypsin (200 ng, Promega, in 25 mM ammonium bicarbonate) at 37°C for 16 h. The extracted peptides were desalted on a home-made C18 desalting tip and concentrated in a SpeedVac (Thermo Savant). Samples were analyzed on a Q Exactive mass spectrometer (Thermo Fisher Scientific) coupled to an Ultimate 3000 nanoRSLC system (Dionex). A 25 cm × 75 μm diameter column was used at a flow rate of 300 nL/min using a 125-min gradient. Data were analyzed with Mascot (version 2.4, Matrix Science, London) against the *Arabidopsis thaliana* proteome database from UniProt. The search criteria were as follows: two missed trypsin cleavage sites allowed; fixed modification carbamidomethyl (C); and variable modification oxidation (M). Precursor mass tolerance was set at +/−20 ppm and fragment mass tolerance at +/−0.02 Da.

## Supplementary Material

Supplementary_Figs_S1-S10_pcag006

Supplementary_Tables_S1-S5_pcag006

Supplementary_Table_S6_pcag006

Supplementary_Table_S7_pcag006

Supplementary_Table_S8_pcag006

## Data Availability

The data underlying this article are available in the article and in its online supplementary materials. The raw mass spectrometry data from the FLAG-based affinity purification of the FTSH4-interacting proteins have been deposited in the MassIVE repository under the title “FLAG-based Affinity Purification and LC-MS/MS Analysis of FTSH4-Interacting Partners in *A. thaliana* Mitochondria” with the dataset identifier MSV000099719. Data related to protein cross-linking and coimmunoprecipitation of SLP1-interacting proteins have been deposited in the Zenodo repository (zenodo.org, DOI number: 10.5281/zenodo.17407310).
