# Supplementary material for: Functional FTSH4 complexes in Arabidopsis mitochondria: a megacomplex with SLP1 and SLP1-free smaller complexes
Source: Plant Cell Physiol. 2026 Feb 19;67(6):872–83. doi: 10.1093/pcp/pcag006 (PMC13365137; doi:10.1093/pcp/pcag006)
Supplement: Supplementary_Figs_S1-S10_pcag006 [file supplementary_figs_s1-s10_pcag006.pdf]

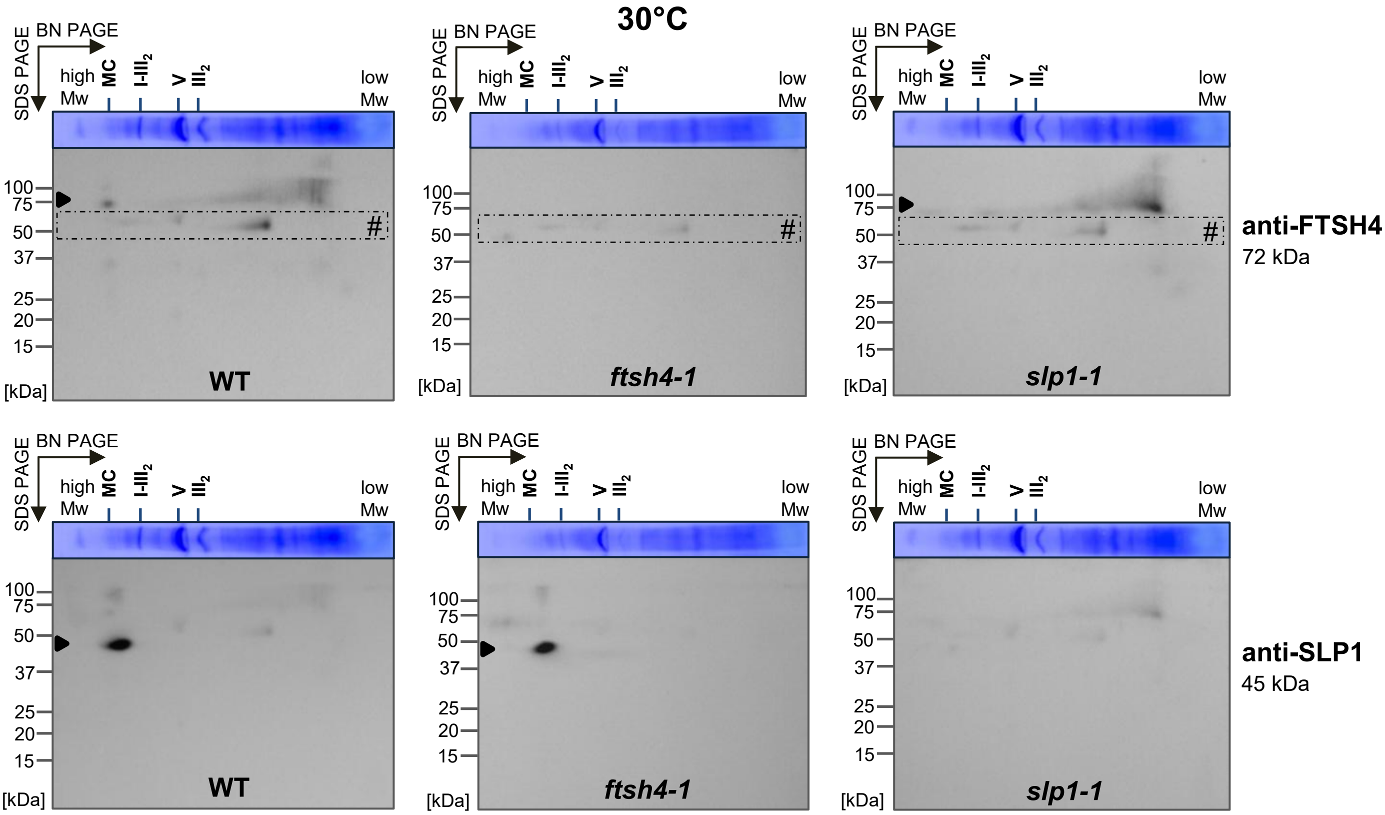


**Figure S1.** Identification of monomeric FTSH4 and SLP1 proteins in *A. thaliana* mitochondria by immunoblotting after BN/SDS-PAGE. Mitochondria were isolated from 2-week-old WT, *ftsh4-1*, and *slp1-1* plants grown at 30°C. After solubilization of mitochondria with digitonin (5 g/1 g mitochondrial protein) and separation of protein complexes by BN-PAGE, the second-dimension electrophoresis was run in a two-step Tricine-SDS gel (8% and 10%) followed by immunodetection of FTSH4 and SLP1. Representative immunoblots with corresponding BN-PAGE gel lanes are shown. The positions of several OXPHOS complexes as well as the FTSH4-SLP1 megacomplex (MC) are indicated on the top of the BN-PAGE gel lane, and the sizes of the molecular weight markers are on the left side of the immunoblots. Black arrowheads mark the position of monomeric forms of FTSH4 (top panel) and SLP1 (bottom panel). # nonspecific immunoblot signals.

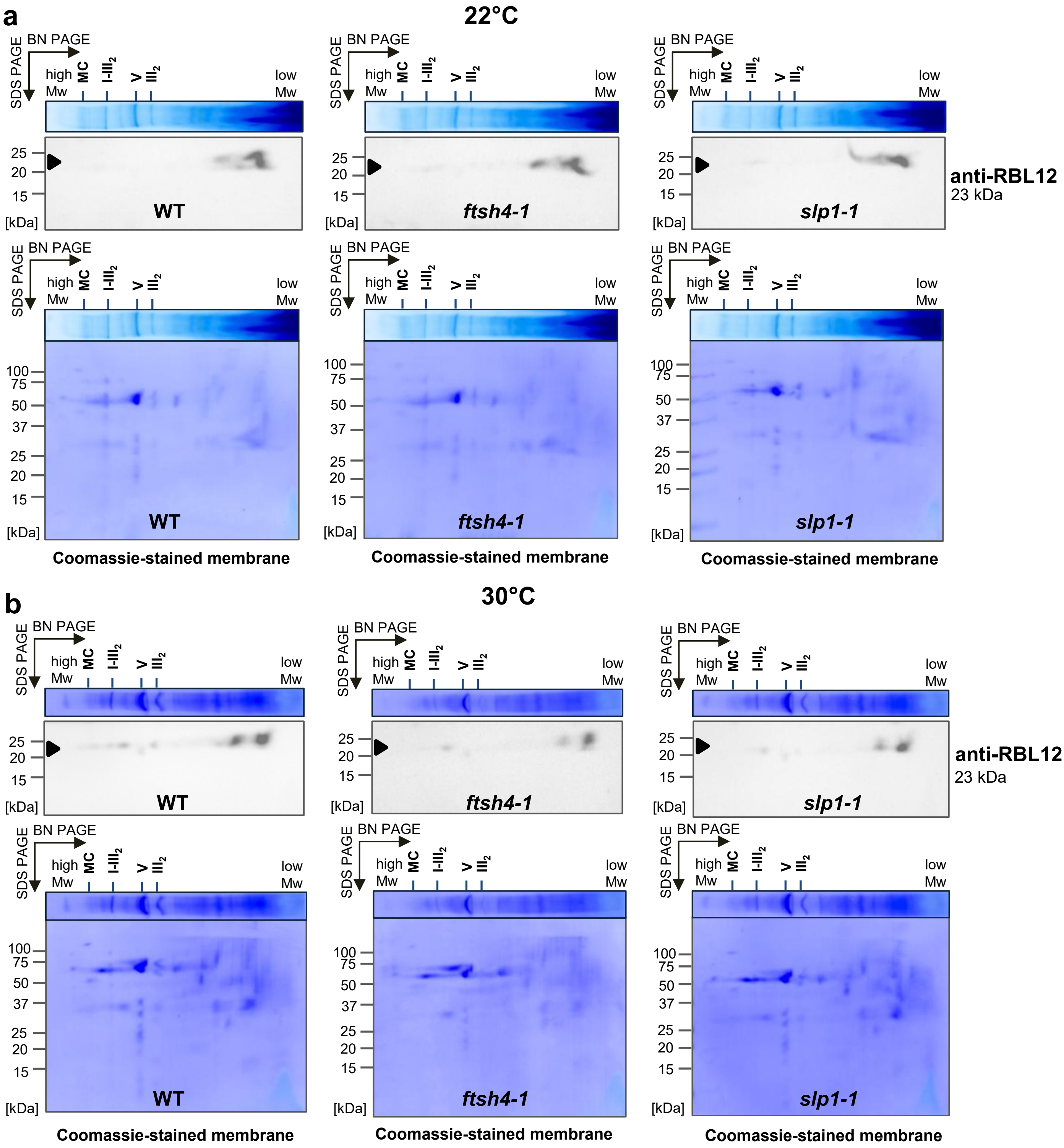
a
22°C
BN PAGE
SDS PAGE
high Mw
MC
I-III2
>
III2
low Mw
25
20
15
[kDa]
WT
ftsh4-1
slp1-1
anti-RBL12
23 kDa
100
75
50
37
25
20
15
[kDa]
Coomassie-stained membrane
b
30°C
BN PAGE
SDS PAGE
high Mw
MC
I-III2
>
III2
low Mw
WT
ftsh4-1
slp1-1
anti-RBL12
23 kDa
Coomassie-stained membrane

**Figure S2.**

**Figure S2.** Identification of monomeric RBL12 protein in *A. thaliana* mitochondria by immunoblotting after BN/SDS-PAGE. Mitochondria were isolated from 2-week-old WT, *ftsh4-1*, and *slp1-1* plants grown at 22°C **(a)** or 30°C **(b)**. After solubilization of mitochondria with digitonin (5 g/1 g mitochondrial protein) and separation of protein complexes by BN-PAGE, the second-dimension electrophoresis was run in a two-step (8% and 10%) Tricine-SDS gel followed by immunodetection of RBL12. Representative immunoblots with corresponding BN-PAGE gel lanes are shown. The positions of several OXPHOS complexes as well as the FTSH4-SLP1 megacomplex (MC) are indicated on the top of the BN-PAGE gel lane, and the sizes of the molecular weight markers are on the left side of the immunoblots. Black arrowheads mark the position of monomeric forms of RBL12.

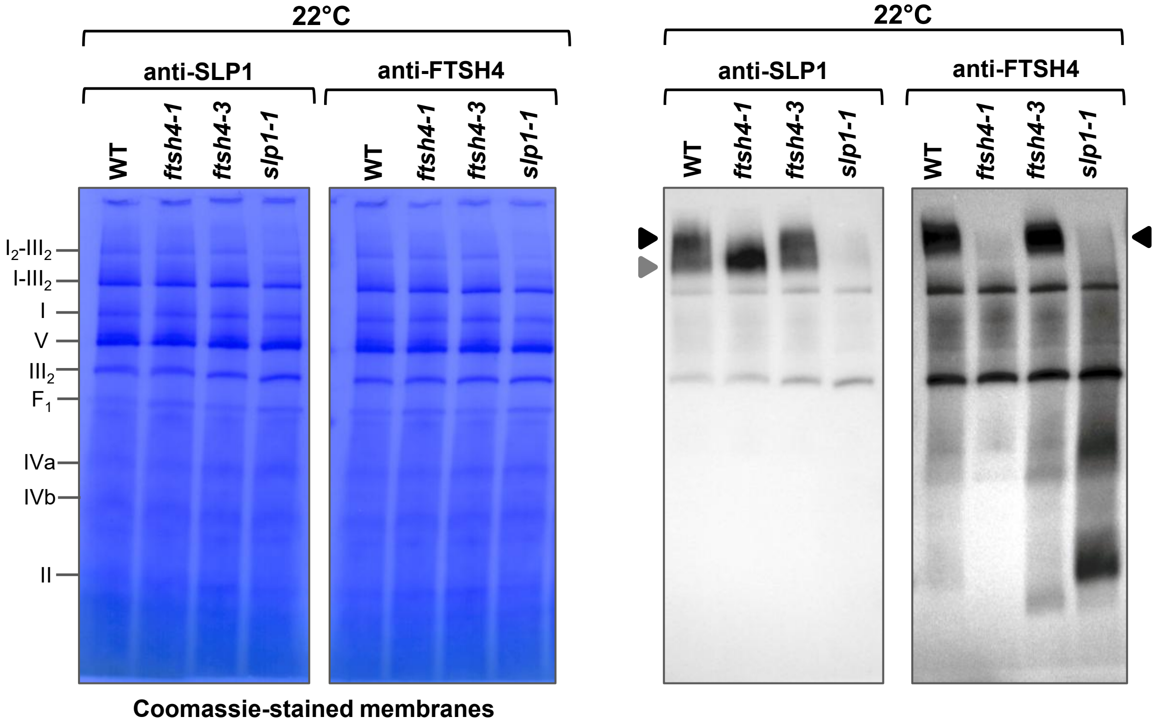


**Figure S3.** FTSH4 complexes in the mitochondria of *A. thaliana ftsh4-3* mutant expressing truncated FTSH4 lacking the plant-specific alanine-rich C-terminus identified by immunoblotting after BN-PAGE. Mitochondria were isolated from 2-week-old WT, *ftsh4-1*, *ftsh4-3*, and *slp1-1* plants grown at 22°C. Purified mitochondria were solubilized with digitonin (5 g/1 g mitochondrial proteins), and proteins were separated by 3-12% BN-PAGE followed by immunodetection of the FTSH4 and SLP1 proteins. Representative immunoblots and Coomassie-stained membranes are shown. Black arrowheads - FTSH4-SLP1 megacomplex; grey arrowheads - FTSH4-free, SLP1-containing complex. OXPHOS complexes indicated on the left served as molecular weight markers. Their molecular weights were assigned following Eubel et al. (2003) and Braun (2020) as follows: $I_2$-$III_2$: 2500 kDa, I-$III_2$: 1500 kDa, I: 1000 kDa, V: 650 kDa, $III_2$: 500 kDa, $F_1$: 350 kDa, IVa: 300 kDa, IVb: 220, II: 160 kDa.

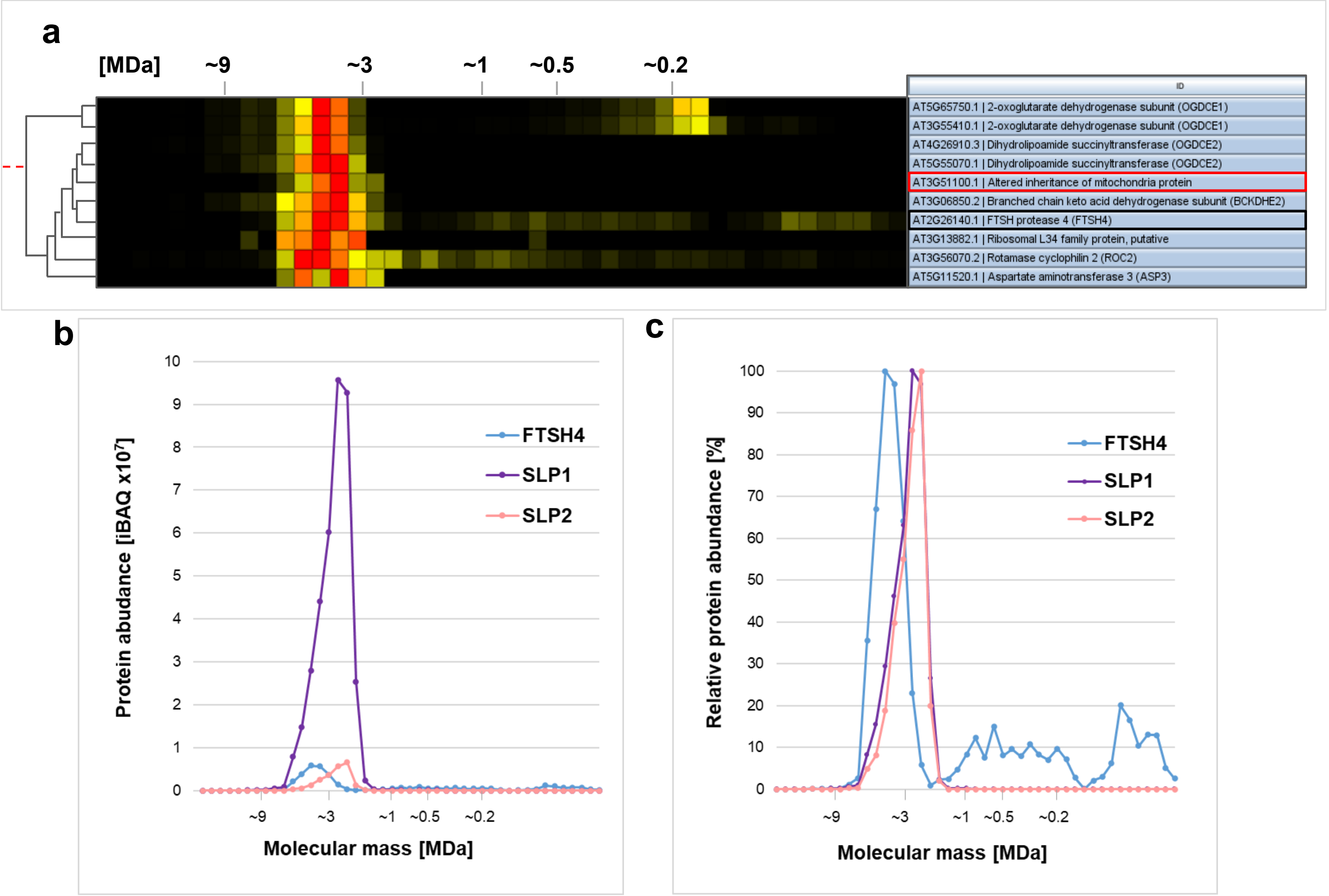


**Figure S4.** Hierarchical clustering of complexome profiling data published by Rugen et al. (2019) for cross-linked WT *A. thaliana* leaf mitochondria. **(a)** Heat maps of the relative abundances of proteins grouping with FTSH4. The panel displays the region near FTSH4 of the master heat map that groups all the proteins. The red dashed line (left site of the clustering image) indicates the branching cutoff point. Highlighted in red is the plant-specific protein AT3G51100 of an unknown function. FTSH4 is highlighted in black. Protein abundance profiles were generated using the NOVA software (Giese et al., 2015). The colours on the heat maps indicate protein abundance (black, no signal or very low abundance; olive-green, low abundance; yellow, medium abundance; orange and red, high or very high abundance). **(b)** and **(c)** Migration profiles of FTSH4, SLP1, and SLP2 in lpBN-PAGE without (B) and with normalization (C). In the normalization process, the quantity of each protein in the richest fraction was set to 100%. iBAQ, intensity-based absolute quantification.

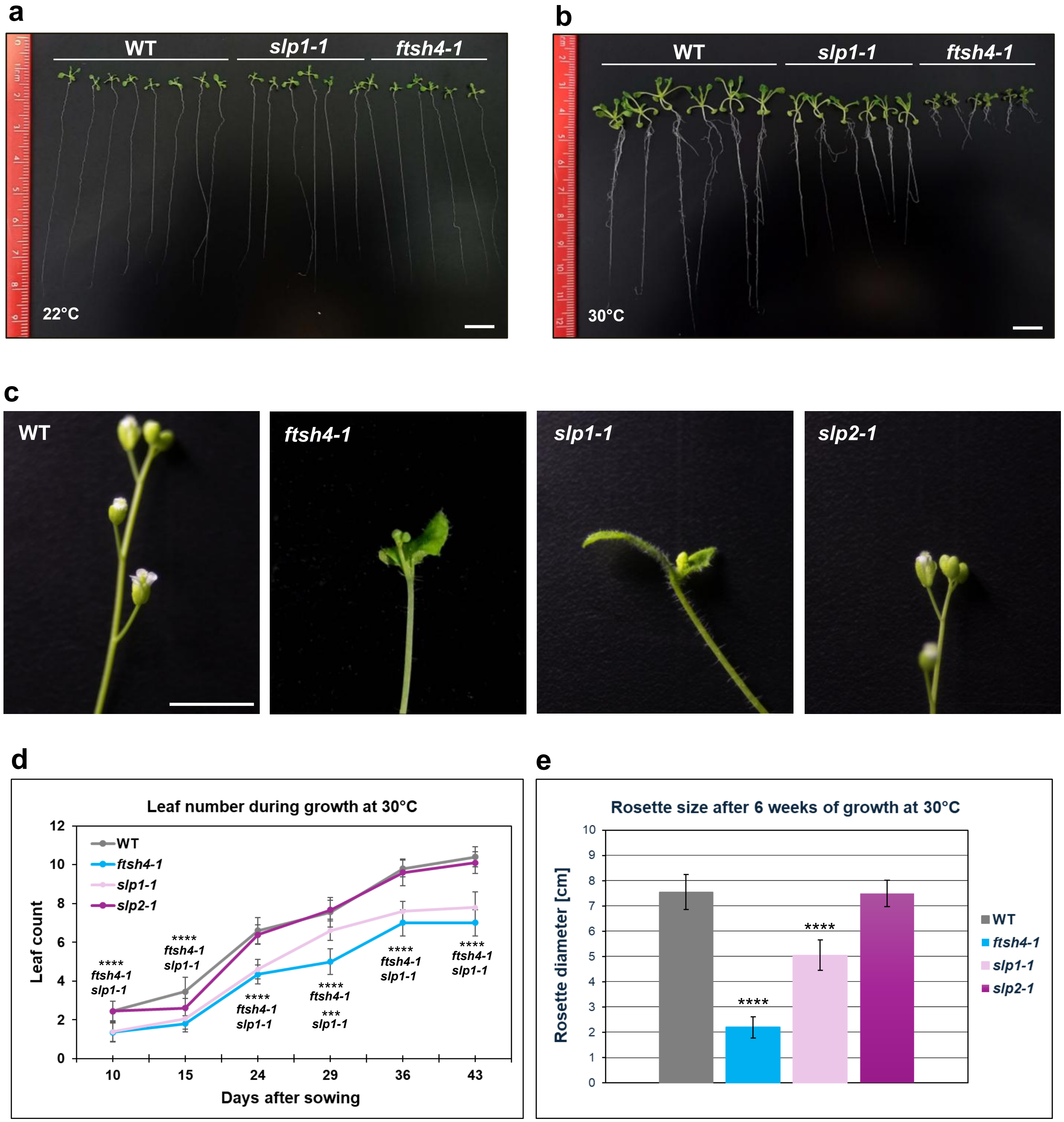


**Figure S5.** Morphology and development of WT, *ftsh4-1*, and *slp* plants. **(a)** and **(b)** WT, *ftsh4-1*, and *slp1-1* seedlings were grown for 2 weeks on 0.5 x MS medium supplemented with 1.5% sucrose and 1% agar at 22ºC **(a)** and 30ºC **(b)**. Scale bars indicate 1 cm. **(c)** Inflorescence stems of six-week-old plants grown in soil at 30ºC. Scale bar indicates 1 cm. **(d)** Number of rosette leaves >1 mm long produced over a period of six weeks in WT, *ftsh4-1*, *slp1-1*, and *slp2-1* plants grown in soil at 30ºC. Data represents mean ± SD, ***$p \leq 0.0001$; ****$p \leq 0.00001$; n >10. **(e)** Size of six-week-old rosettes from WT, *ftsh4-1*, *slp1-1*, and *slp2-1* plants grown in soil at 30ºC. Data represents mean ± SD, ****$p \leq 0.00001$; n=10.

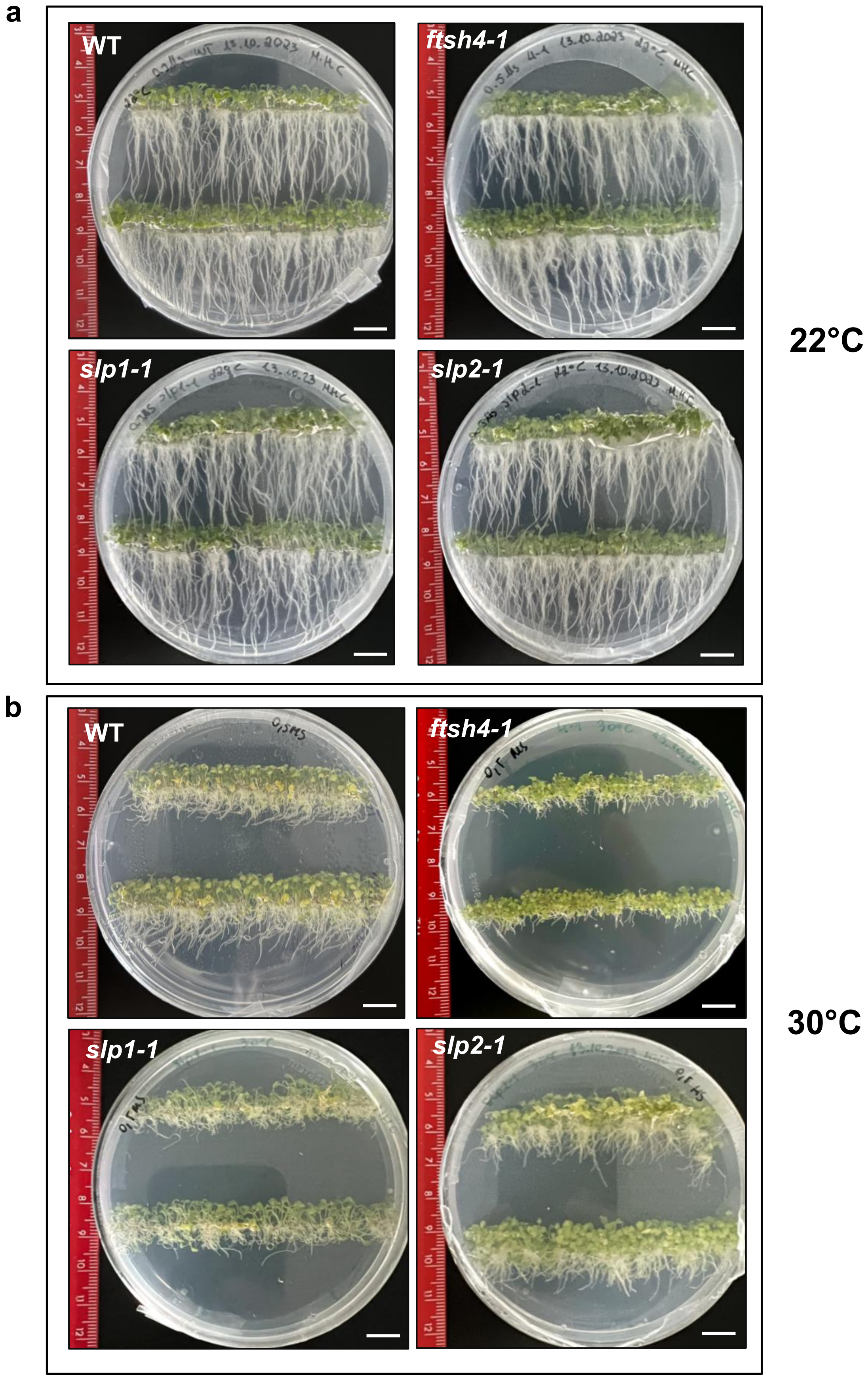


**Figure S6.** Morphology of WT, *ftsh4-1*, *slp1-1*, and *slp2-1* plants. Seedlings were grown for 7 days on 0.5 x MS medium supplemented with 1.5% sucrose at 22ºC (**a**) and 30ºC (**b**). Scale bars indicate 1 cm.

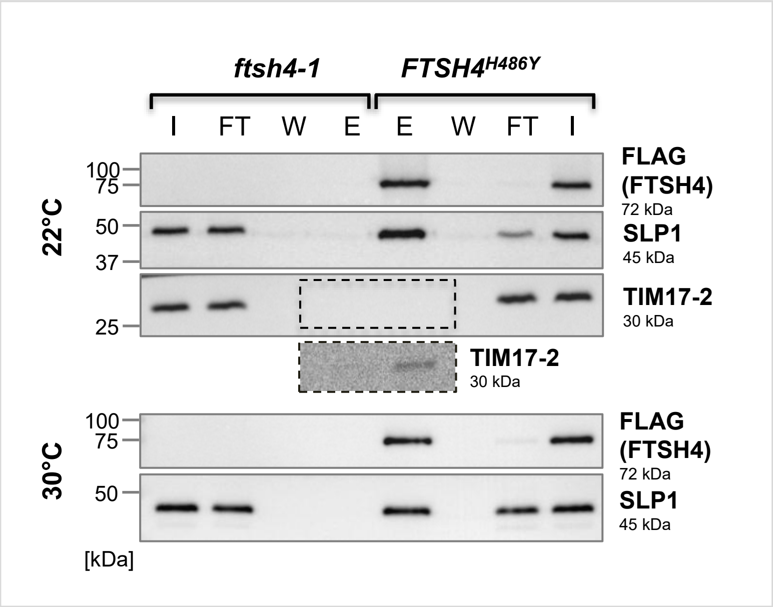


**Figure S7.** Identification of FTSH4-interacting proteins. Mitochondria were isolated from the *FTSH4*$^{H486Y}$ and *ftsh4-1* mutants grown at 22ºC and 30ºC, and subjected to FLAG-based affinity purification. The *ftsh4-1* mutant mitochondria were used as a negative control. Immunodetection was performed on gel-separated samples collected at various stages throughout the experiment: IN, input; FL, flow-through; W, wash; E, eluate. Anti-FLAG antibodies were used to detect the FTSH4-interacting proteins and to control the accuracy of co-IP. Anti-SLP1 and anti-TIM17-2 antibodies were used to confirm previously detected FTSH4-interacting proteins (Tim17-2; Opalinska et al., 2018; SLP1, Opalinska et al., 2017).

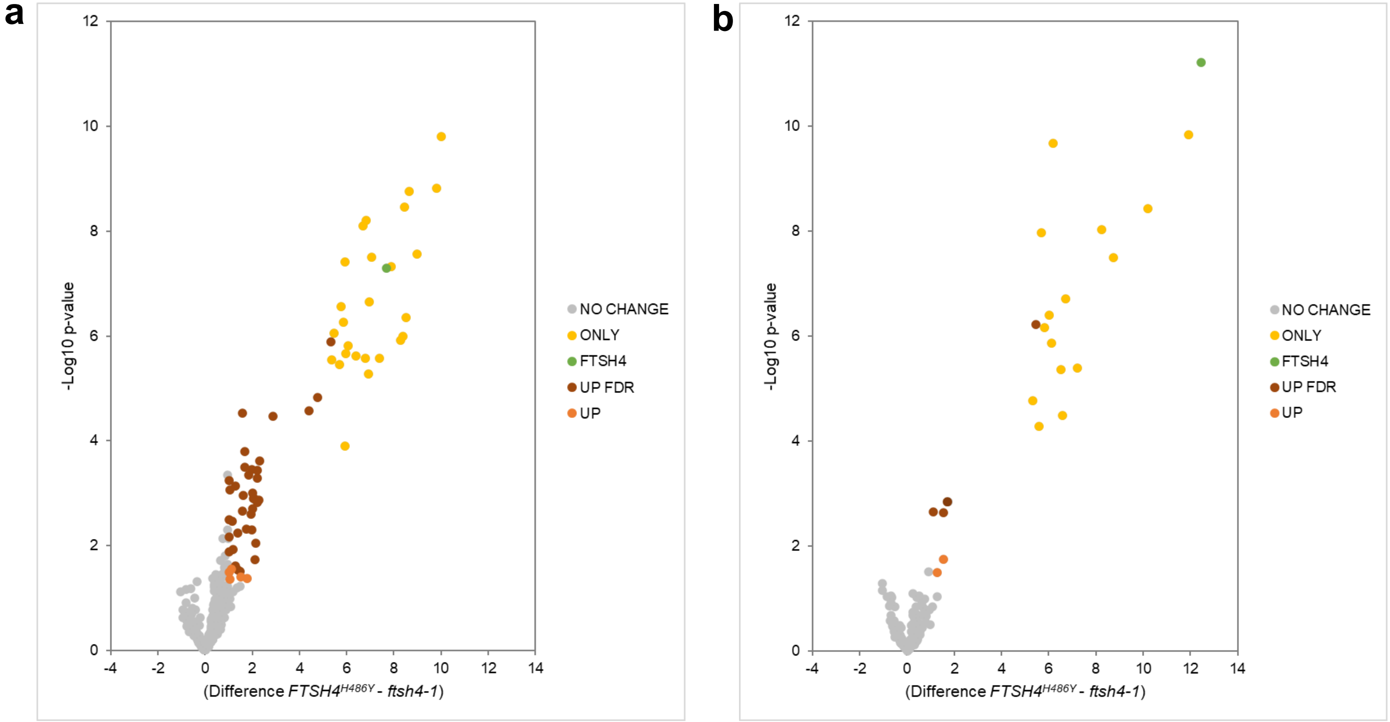


**Figure S8.** Statistical evaluation of selected FTSH4-interacting proteins. The volcano plot illustrates the distribution of proteins identified in proteomic analyses aimed at detecting potential interacting partners of the FTSH4 protease at **(a)** 22°C and **(b)** 30°C. The x-axis shows the $log_2$ fold change in protein abundance between the *FTSH4*$^{H486}$ sample and the *ftsh4-1* control sample, while the y-axis shows the -$log_{10}$ (*p*-value) indicating statistical significance. Proteins that are significantly enriched in the *FTSH4*$^{H468Y}$ samples ($p \leq 0.05$; $log_2$ FC ≥1) are colored and represent potential FTSH4 interactors. Non-significant proteins are shown in grey (NO CHANGE). Yellow: protein identified only in *FTSH4*$^{H486Y}$ samples (ONLY); Green: FTSH4 protein; Brown: protein with FDR < 0.05 (UP FDR); Orange: protein with FDR > 0.05 (UP).

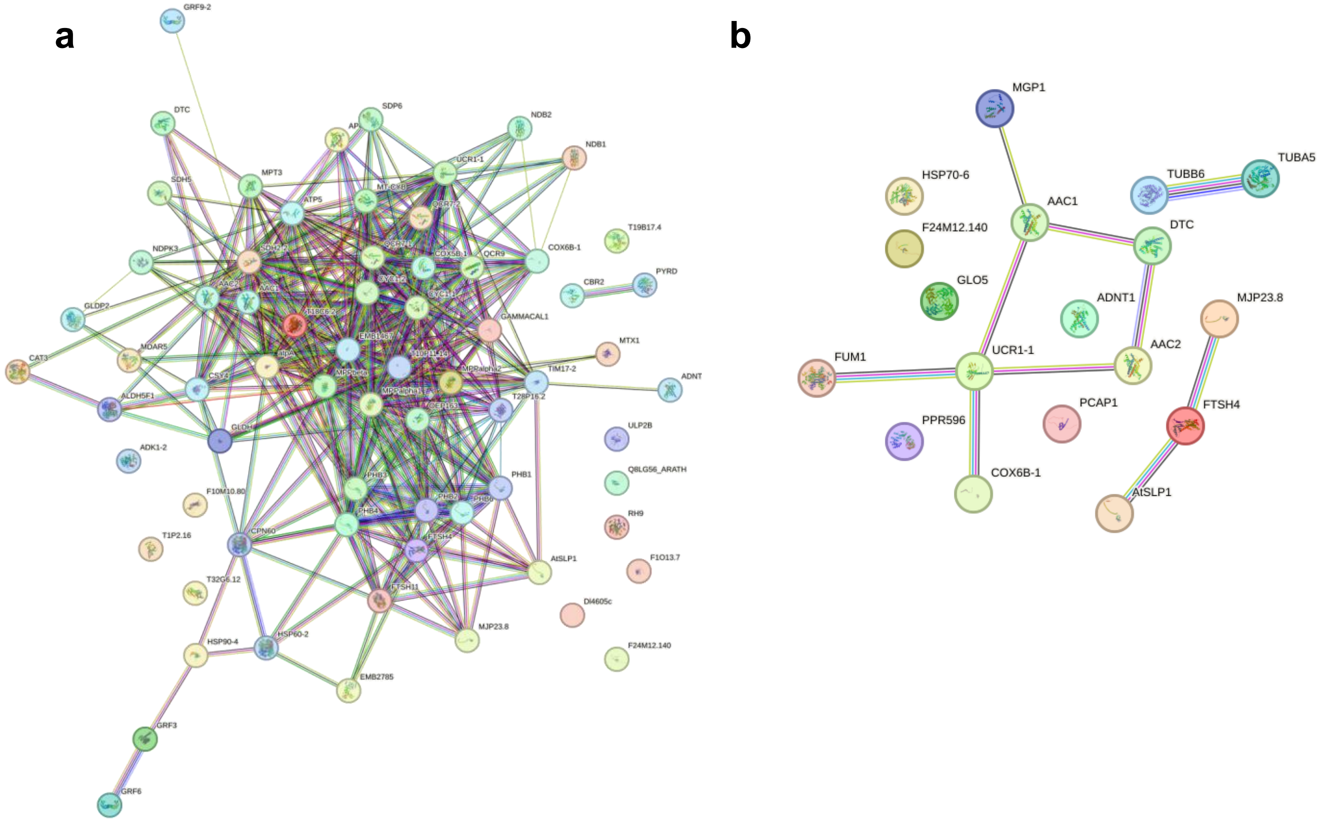


**Figure S9.** STRING analysis of FTSH4-interacting proteins. The graphical representation of potential interactions between proteins, based on STRING analysis for selected FTSH4-interactors at **(a)** 22°C and **(b)** 30°C. Links to the full analysis are provided below.

**(a)** 22°C https://version-12-0.string-db.org/cgi/network?networkId=b7uG9KWXbvoj

**(b)** 30°C https://version-12-0.string-db.org/cgi/network?networkId=bqEQSiNcmkSe

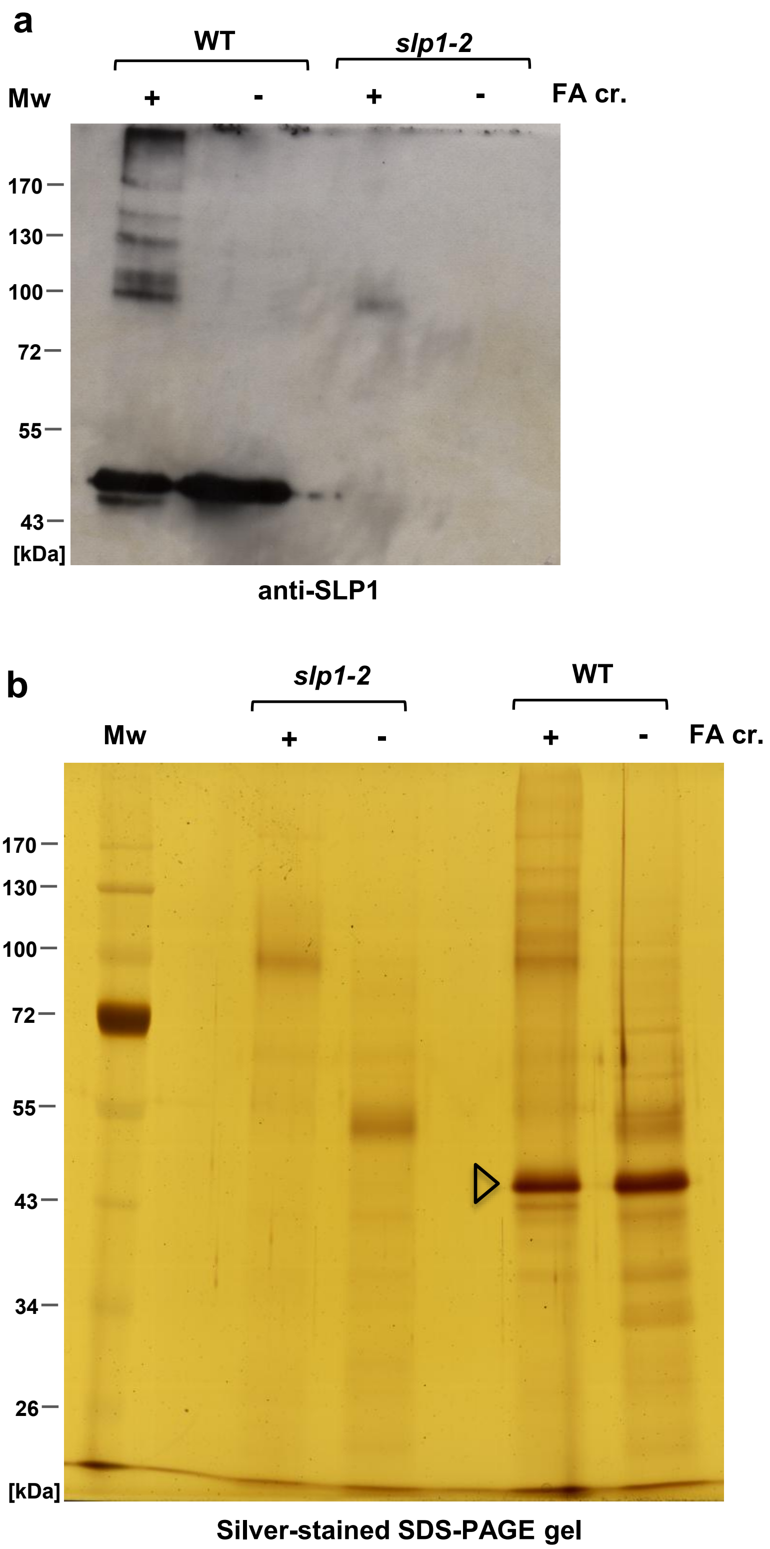


**Figure S10.** Identification of SLP1-interacting proteins. **(a)** Western blot (anti-SLP1) of immunoprecipitated SLP1 from wild-type (WT, *qrt1-2)* or *slp1-2* mitochondria. "+ FA cr." indicates crosslinked sample, "- FA cr." indicates reversal of FA crosslinking by heating. Mw = molecular weight marker in kDa. **(b)** Silver-stained SDS-PAGE gel of immunoprecipitated fractions. Labelling of samples as above. The white arrow indicates the position of SLP1.
