# Supplementary material for: Functional FTSH4 complexes in Arabidopsis mitochondria: a megacomplex with SLP1 and SLP1-free smaller complexes
Source: Plant Cell Physiol. 2026 Feb 19;67(6):872–83. doi: 10.1093/pcp/pcag006 (PMC13365137; doi:10.1093/pcp/pcag006)
Supplement: Supplementary_Tables_S1-S5_pcag006 [file supplementary_tables_s1-s5_pcag006.xlsx]

**Supplementary Tables**

**Table S1**. **Proteins identified by MS/MS as FTSH4-interactors in *FTSH4^H486Y^* mitochondria isolated from plants grown at 22°C.** Mitochondria isolated from *ftsh4-1* mutant were used as a control. Proteins are ordered according to Mascot Score. “No. Peptides” indicates the number of identified peptides; “No. PSMs” = number of peptide-to-spectrum matches; “Seq. Cov.” = sequence coverage in %. AGI, Arabidopsis Genome Initiative Identifier (TAIR). UniProt, Universal Protein Resource database.

| **No** | **AGI** | **UniProt** | **Name** | **Mascot Score** | **No. Peptides** | **No. PSMs** | **% Seq. Cov.** |
| --- | --- | --- | --- | --- | --- | --- | --- |
| 1. | At2g26140 | O80983 | FTSH4 | 14690.26 | 54 | 2002 | 72.66 |
| 2. | At4g27585 | Q93VP9 | SLP1 | 6995.33 | 34 | 1009 | 76.64 |
| 3. | At5g54100 | Q9LVW0 | SLP2 | 4135.67 | 22 | 569 | 56.61 |
| 4. | At3g51100 | Q9SD36 | F24M12.140 | 1675.54 | 14 | 219 | 62.56 |
| 5. | At3g02090 | Q42290 | MPPbeta | 1458.30 | 24 | 245 | 53.67 |
| 6. | At3g23990 | P29197 | HSP60; CPN60 | 1374.68 | 33 | 188 | 64.64 |
| 7. | At3g08580 | P31167 | AAC1 | 1302.09 | 20 | 194 | 39.90 |
| 8. | At1g51980 | Q9ZU25 | MPPalpha1 | 1271.25 | 23 | 226 | 56.26 |
| 9. | At2g33210 | F4IVR2 | HSP60-2 | 1160.20 | 28 | 159 | 56.90 |
| 10. | At2g07698 | F4IMB5 | ATP synthase subunit alpha | 1040.84 | 22 | 168 | 29.73 |
| 11. | At1g20620 | Q42547 | CAT3 | 910.29 | 22 | 168 | 47.76 |
| 12. | At5g13490 | P40941 | AAC2 | 727.97 | 14 | 104 | 31.17 |
| 13. | At3g16480 | O04308 | MPPalpha2 | 524.25 | 14 | 88 | 30.06 |
| 14. | At1g22450 | Q9S7L9 | COX6B-1 | 459.36 | 10 | 70 | 53.93 |
| 15. | At5g13430 | Q94JS0 | UCR1-1 | 420.95 | 6 | 64 | 26.84 |
| 16. | At5g40770 | O04331 | PHB3 | 420.70 | 13 | 65 | 58.12 |
| 17. | At4g04320 | F4JGB9 | AT4G04320 | 406.10 | 20 | 67 | 43.91 |
| 18. | At5g38480 | P42644 | GRF3 | 399.15 | 4 | 72 | 14.51 |
| 19. | At4g01100 | O04619 | ADNT1 | 398.36 | 15 | 67 | 45.17 |
| 20. | At5g10450 | F4KGV2 | GRF6 | 375.74 | 3 | 63 | 10.57 |
| 21. | At3g27280 | Q9LK25 | PHB4 | 356.13 | 9 | 48 | 34.41 |
| 22. | At3g27240 | Q9LK29 | CYC1-1 | 347.87 | 10 | 76 | 46.58 |
| 23. | At2g42590 | F4IP53 | GRF9 | 343.32 | 2 | 54 | 6.87 |
| 24. | At3g09820 | O82514 | ADK1 | 339.71 | 11 | 59 | 50.00 |
| 25. | At5g19760 | Q9C5M0 | DTC | 327.86 | 8 | 57 | 32.21 |
| 26. | At5g40810 | Q9FKS5 | CYC1-2 | 323.22 | 10 | 65 | 46.58 |
| 27. | At5g37510 | B9DFQ9 | 75 kDa; EMB1467 | 322.85 | 20 | 65 | 31.81 |
| 28. | At4g11010 | O49203 | NDPK3 | 290.36 | 9 | 49 | 38.66 |
| 29. | At4g32470 | Q9SUU5 | QCR7-1 | 249.97 | 7 | 48 | 50.82 |
| 30. | At3g47930 | Q9SU56 | GLDH | 245.86 | 17 | 55 | 31.80 |
| 31. | At1g03860 | Q9ZNT7 | PHB2 | 215.25 | 9 | 34 | 46.85 |
| 32. | At5g14040 | Q9FMU6 | PHT3;1; MPT3 | 211.55 | 11 | 46 | 31.47 |
| 33. | At2g42210 | O48528 | B14.7 | 199.89 | 6 | 39 | 50.94 |
| 34. | At3g10370 | Q9SS48 | SDP6 | 197.35 | 14 | 34 | 31.16 |
| 35. | At1g47420 | Q9SX77 | SDH5 | 190.65 | 5 | 28 | 35.41 |
| 36. | At4g28510 | O49460 | PHB1 | 185.79 | 10 | 29 | 47.57 |
| 37. | At5g53170 | Q9FGM0 | FTSH11 | 185.49 | 3 | 47 | 3.72 |
| 38. | At2g20530 | Q9SIL6 | PHB6 | 171.77 | 7 | 35 | 31.47 |
| 39. | At3g15640 | A8MRD7 | At3g15640, COX5B-1 | 169.92 | 5 | 38 | 22.29 |
| 40. | At5g13450 | Q96251 | ATP5 | 165.52 | 8 | 26 | 40.76 |
| 41. | At2g26080 | O80988 | GLDP2 | 162.33 | 10 | 26 | 8.05 |
| 42. | At1g79440 | Q9SAK4 | ALDH5F1 | 154.41 | 9 | 21 | 20.45 |
| 43. | At5g23300 | P32746 | PYRD | 147.27 | 10 | 22 | 24.57 |
| 44. | At4g17140 | F4JNE3 | AT4G17140 | 140.18 | 18 | 29 | 4.95 |
| 45. | AtMg00220 | A0A2P2CLG2 | MT-CYB, COB | 138.45 | 2 | 15 | 5.60 |
| 46. | At3g52730 | Q9LXJ2 | QCR9 | 137.47 | 3 | 18 | 31.94 |
| 47. | At3g22310 | A0A1I9LT07 | PMH1 | 136.13 | 12 | 27 | 23.03 |
| 48. | At4g05020 | Q94BV7 | NDB2 | 132.32 | 10 | 20 | 18.38 |
| 49. | At5g63510 | Q9FMV1 | GAMMA CAL1 | 130.74 | 5 | 19 | 21.83 |
| 50. | At4g28220 | Q1JPL4 | NDB1 | 120.48 | 10 | 21 | 20.14 |
| 51. | At1g05205 | Q8LG56 | AT1G05205 | 120.41 | 4 | 24 | 39.56 |
| 52. | At2g04940 | Q9SI32 | AT2G04940 | 114.72 | 5 | 17 | 14.29 |
| 53. | At2g19080 | O64471 | MTX1 | 105.95 | 5 | 14 | 22.54 |
| 54. | At2g37410 | Q9SP35 | TIM17-2 | 104.04 | 3 | 21 | 21.40 |
| 55. | At4g02580 | O22769 | 24 kDa, AT4G02580 | 103.69 | 5 | 27 | 22.35 |
| 56. | At2g30970 | P46643 | ASP1 | 101.65 | 6 | 21 | 15.81 |
| 57. | At5g40650 | Q8LB02 | SDH2-2 | 90.69 | 5 | 15 | 17.86 |
| 58. | At4g34310 | F4JKN4 | AT4G34310 | 85.73 | 11 | 15 | 10.99 |
| 59. | At5g20080 | P83291 | CBR2 | 79.13 | 9 | 19 | 34.45 |
| 60. | At2g44350 | P20115 | ATCS; CSY4 | 78.37 | 8 | 20 | 20.89 |
| 61. | At2g31490 | Q9SIQ8 | AT2G31490 | 75.44 | 3 | 17 | 43.66 |
| 62. | At1g29960 | Q67XF2 | IMP1-2 | 71.74 | 3 | 8 | 18.34 |
| 63. | At5g25450 | F4JWS9 | QCR7-2 | 68.21 | 2 | 14 | 16.04 |
| 64. | At1g63940 | F4I577 | MDAR6 | 62.27 | 4 | 7 | 12.74 |
| 65. | AtCg00120 | P56757 | atpA | 61.63 | 2 | 9 | 3.94 |
| 66. | At5g56000 | O03986 | Hsp81.4 | 56.66 | 2 | 6 | 3.15 |
| 67. | At2g41600 | A8MSD8 | AT2G41600 | 56.47 | 3 | 8 | 22.22 |
| 68. | At5g51050 | Q9FI43 | APC2 | 46.73 | 5 | 9 | 9.03 |
| 69. | At2g31060 | F4IPW4 | EMB2785 | 43.49 | 6 | 10 | 11.24 |

**Table S2**. **Proteins identified by MS/MS as FTSH4-interactors in *FTSH4^H486Y^* mitochondria isolated from plants grown at 30°C.** Mitochondria isolated from *ftsh4-1* mutant were used as a control. Proteins are ordered according to Mascot Score. “No. Peptides” indicates the number of identified peptides; “No. PSMs” = number of peptide-to-spectrum matches; “Seq. Cov.” = sequence coverage in %. AGI. Arabidopsis Genome Initiative Identifier (TAIR). UniProt. Universal Protein Resource database.

| **No.** | **AGI** | **UniProt** | **Name** | **Mascot Score** | **No. Peptides** | **No. PSMs** | **% Seq. Cov.** |
| --- | --- | --- | --- | --- | --- | --- | --- |
| 1. | At4g27585 | Q93VP9 | SLP1 | 6078.28 | 31 | 874 | 76.16 |
| 2. | At2g26140 | O80983 | FTSH4 | 5702.70 | 43 | 811 | 67.64 |
| 3. | At5g54100 | Q9LVW0 | SLP2 | 3937.56 | 19 | 533 | 54.11 |
| 4. | At3g51100 | Q9SD36 | F24M12.140 | 1201.57 | 11 | 156 | 60.19 |
| 5. | At3g08580 | P31167 | AAC1 | 632.48 | 15 | 91 | 37.27 |
| 6. | At5g13490 | P40941 | AAC2 | 447.51 | 10 | 63 | 25.45 |
| 7. | At4g04320 | F4JGB9 | T19B17.4 | 442.81 | 16 | 78 | 37.72 |
| 8. | At5g44780 | A0A1R7T3H8 | MORF4 | 313.13 | 15 | 61 | 30.99 |
| 9. | At1g22450 | Q9S7L9 | COX6B-1 | 273.77 | 6 | 38 | 34.03 |
| 10. | At5g13430 | Q94JS0 | UCR1-1 | 170.17 | 7 | 31 | 26.84 |
| 11. | At4g18360 | O49506 | GOX3; GLO5 | 119.84 | 4 | 17 | 10.33 |
| 12. | At1g07930 | F4HUA0 | T6D22.31;  EF-1-alpha 2 | 111.67 | 3 | 15 | 8.06 |
| 13. | At5g19760 | Q9C5M0 | DTC | 108.72 | 5 | 14 | 20.13 |
| 14. | At4g01100 | O04619 | ADNT1 | 106.00 | 8 | 19 | 26.42 |
| 15. | At5g19780 | B9DHQ0 | TUA5 | 73.88 | 2 | 10 | 6.22 |
| 16. | At5g12250 | P29514 | TUB6 | 73.66 | 2 | 9 | 5.79 |
| 17. | At2g21870 | Q9SJ12 | MGP1 | 68.24 | 5 | 8 | 23.75 |
| 18. | At3g15640 | A8MRD7 | COX5B-1 | 64.68 | 5 | 16 | 22.29 |
| 19. | At2g47510 | P93033 | FUM1 | 61.25 | 6 | 10 | 14.63 |
| 20. | At1g80270 | Q9C977 | PPR596 | 59.72 | 6 | 11 | 10.91 |
| 21. | At4g20260 | Q96262 | PCAP1 | 52.47 | 3 | 7 | 16.00 |
| 22. | At4g24280 | Q9STW6 | HSP70-6; CPHSC70-1 | 46.52 | 5 | 8 | 7.94 |

**Table S3**. **Proteins identified by MS/MS as SLP1-interactors in *qrt1-2* mitochondria.** Mitochondria isolated from *slp1-2* mutant were used as a control. Proteins are ordered according to Mascot Score. “No. Peptides” indicates the number of significant peptide matches in each hit; “No. DS” = number of distinct significant sequences per hit; “% Seq. Cov.” = sequence coverage in %; emPAI is a rough indicator of protein abundance. AGI. Arabidopsis Genome Initiative Identifier (TAIR). UniProt. Universal Protein Resource database.

| **No.** | **AGI** | **UniProt** | **Name** | **Mascot Score** | **No. Peptides** | **No. DS** | **% Seq. Cov.** | **emPAI** |
| --- | --- | --- | --- | --- | --- | --- | --- | --- |
| 1 | At4g27585 | Q93VP9 | SLP1 | 1268 | 59 | 31 | 72 | 13.68 |
| 2 | At5g54100 | Q9LVW0 | SLP2 | 498 | 20 | 11 | 26 | 1.58 |
| 3 | At5g08680 | Q9C5A9 | ATP synthase subunit beta-3 | 193 | 6 | 6 | 13 | 0.38 |
| 4 | At4g37930 | Q9SZJ5 | SHM1 | 141 | 6 | 5 | 11 | 0.4 |
| 5 | At2g26140 | O80983 | FTSH4 | 98 | 6 | 6 | 11 | 0.28 |
| 6 | At2g33210 | F4IVR2 | HSP60-2 | 95 | 5 | 4 | 11 | 0.23 |
| 7 | AtMg01190 | P92549 | ATPA | 93 | 4 | 3 | 7 | 0.19 |
| 8 | At3g23990 | P29197 | CPN60 | 82 | 6 | 6 | 13 | 0.37 |
| 9 | At2g20420 | O82662 | Succinyl-CoA ligase subunit beta | 79 | 2 | 2 | 9 | 0.15 |
| 10 | At2g26080 | O80988 | GLDP2 | 72 | 3 | 3 | 4 | 0.09 |
| 11 | At5g08300 | P68209 | Succinyl-CoA ligase subunit alpha-1 | 72 | 1 | 1 | 3 | 0.09 |
| 12 | At2g18450 | Q9ZPX5 | SDH1-2 | 68 | 1 | 1 | 2 | 0.05 |
| 13 | At1g53240 | Q9ZP06 | MMDH1 | 68 | 2 | 2 | 9 | 0.19 |
| 14 | At4g26970 | Q94A28 | ACO2 | 55 | 2 | 2 | 2 | 0.06 |
| 15 | At5g09590 | Q9LDZ0 | HSP70-10 | 55 | 3 | 2 | 5 | 0.14 |
| 16 | At5g46180 | Q9FNK4 | DELTA-OAT | 52 | 1 | 1 | 1 | 0.06 |
| 17 | At4g39660 | Q940M2 | AGT2 | 49 | 3 | 3 | 7 | 0.2 |
| 18 | At3g13930 | Q8RWN9 | MTE2-2 | 47 | 2 | 2 | 4 | 0.11 |
| 19 | At2g44350 | P201152 | CSY4 | 46 | 2 | 2 | 9 | 0.13 |
| 20 | At5g15090 | Q9SMX3 | VDAC3 | 40 | 2 | 2 | 11 | 0.24 |
| 21 | At5g14780 | Q9S7E4 | FDH1 | 39 | 3 | 3 | 7 | 0.25 |
| 22 | At3g62530 | Q94K48 | ARM repeat superfamily protein | 35 | 1 | 1 | 5 | 0.14 |
| 23 | At4g37910 | Q8GUM2 | HSP70-9 | 33 | 2 | 2 | 3 | 0.09 |
| 24 | At5g63400 | O82514 | ADK1 | 32 | 1 | 1 | 6 | 0.12 |
| 25 | At3g06050 | Q9M7T0 | Peroxiredoxin-2F | 31 | 1 | 1 | 5 | 0.16 |
| 26 | At1g49780 | Q9FXA4 | PUB26 | 29 | 1 | 1 | 1 | 0.07 |
| 27 | At3g17240 | Q9M5K2 | LPD2 | 29 | 1 | 1 | 2 | 0.06 |
| 28 | AtMg01275 | Q1ZXW0 | NAD1 | 27 | 1 | 1 | 2 | 0.09 |
| 29 | At1g47260 | Q9C6B3 | GAMMACA2 | 25 | 1 | 1 | 4 | 0.11 |
| 30 | At5g07440 | F4K6P9 | GDH2 | 24 | 1 | 1 | 7 | 0.1 |
| 31 | At3g48000 | Q9SU63 | ALDH2B4 | 23 | 1 | 1 | 2 | 0.06 |

**Table S4.** **Antibodies used in this study.**

All antibodies were polyclonal.

| **Antibody** | **Source** | **Dilution** |
| --- | --- | --- |
| **FTSH4** | Agrisera (AS07 205) | 1:1000 |
| **SLP1** | Gehl et al., 2014 | 1:5000 |
| **IDH** | Agrisera (AS06 203A) | 1:5000 |
| **LETM1** | Kind gift from Olivier van Aken. University of Lund. Sweden  Zhang et al., 2012 | 1:5000 |
| **TOM40** | Kind gift from Monika Murcha. University of Western Australia. Australia  Carrie et al., 2009 | 1:1000 |
| **TIM17-2** | Kind gift from Monika Murcha. University of Western Australia. Australia Murcha et al., 2005 | 1:10000 |
| **HSP23.6** | Agrisera (AS15 2980) | 1:1000 |
| **NAD9** | Kind gift from Jose M. Gualberto and Geraldine Bonnard. CNRS. Strasbourg. France  Lamattina et al., 1993 | 1:5000 |
| **CytC** | Agrisera (AS08 343A) | 1:5000 |
| **RBL12** | Kmiec-Wisniewska et al., 2008 | 1:1000 |

**Table S5.** **Primer pairs used for RT-qPCR.** AGI. Arabidopsis Genome Initiative Identifier (TAIR).

| **AGI** | **Gene** | **Forward Primer** | **Reverse Primer** |
| --- | --- | --- | --- |
| **Reference Gene** | | | |
| At3g18780 | *ACT2* | ATGGCTGAGGCTGATGATATTC | TGTACGACCACTGGCGTAC |
| **Mitochondrial Unfolded Protein Response** | | | |
| At3g22370 | *AOX1A* | ATGATAACTCGCGGTGGAGC | TCGTCGGAGCTCTAGTCCAT |
| At5g51440 | *HSP23.5* | TGGCGTCATCATCGGCTTTAG | TCATAGTTTCTGGCGGCGTT |
| At2g33210 | *HSP60-2* | CCTTCACAGCACAGACCTTGAT | ACTGGCCCTCAAGAAGCAAA |
| At4g37910 | *mtHSC70-1* | AGGTTTGGTGGGCAAGTTGA | CTTGCACTTTGGGAACACGG |
| At5g55200 | *MGE1* | GTCGCTCCGAAACCATGGAAC | GCTTTCCCCACCTCCACATTC |
| At4g05020 | *NDB2* | TGAGAAGGCAAGTCTACCAGAAC | GCACTGAACCCTTGGCTCTA |
| At2g21640 | *UPOX* | ATCATCATGCAGGAAGAGGGTG | GAAGCTCCCGAATATCTTGTCC |
| **Mitochondrial Proteolytic System** | | | |
| At3g03420 | *ATP23* | CATGATTCGTAGGAGTTTCC | TGCAGACAGTTATCCCTCTT |
| At5g23140 | *CLPP2* | CTGAGAATCCTTCGAAGCCGATCC | AAATTGCAAGACCGGCGGTGAC |
| At5g36950 | *DEG10* | TCCGCCGAATCTCCACTTCT | ATTCGCGCTCTGAGAAGAACA |
| At1g07510 | *FTSH10* | CGCTGAGCTCTTGCTAGAGAAG | ATTCCATCATCCTCCACAGGCT |
| At2g29080 | *FTSH3* | CGCCGAGCTTTTACTAGAGAAA | AGCTCCATCATCCACCACAGGC |
| At2g26140 | *FTSH4* | GCTATGGAGTACAATCCGAAC | CTTCTTCGTGCAAACCAAGTC |
| At5g26860 | *LON1* | GTAGGCAAAACTAGTATT | TATCAATCTCATCGATTAG |
| At5g51740 | *OMA1* | GGAGATAGAAGCAGACTACATC | GCCTTCAACGCCAGTACGACC |
| At1g18600 | *RBL12* | TTGTTCTTGGGTTAGTTATAG | TGTCCAATATCAATATGACTG |
| **Other** | | | |
| At4g27585 | *SLP1* | GGGTCTTCAGTGCCTTCGTT | TGTGCTCGATTCACCTGGTC |
